# Temporal unsnarling of brain’s acute neuroinflammatory transcriptional profiles reveals panendothelitis as the earliest event preceding microgliosis

**DOI:** 10.1038/s41380-020-00955-5

**Published:** 2020-12-08

**Authors:** Mahesh Chandra Kodali, Hao Chen, Francesca-Fang Liao

**Affiliations:** 1 Department of Pharmacology, Addiction Science and Toxicology, College of Medicine, University of Tennessee Health Science Center, Memphis, TN 38103 USA; 2 Integrated Biomedical Sciences Program, Molecular and Systems Pharmacology Track, College of Graduate Health Sciences, University of Tennessee Health Science Center, Memphis, TN 38163 USA

**Keywords:** Neuroscience, Psychiatric disorders

## Abstract

Sepsis-associated encephalopathy (SAE) is an acutely progressing brain dysfunction induced by systemic inflammation. The mechanism of initiation of neuroinflammation during SAE, which ultimately leads to delirium and cognitive dysfunction, remains elusive. We aimed to study the molecular events of SAE to capture its onset and progression into the central nervous system (CNS), and further identify the cellular players involved in mediating acute inflammatory signaling. Gene expression profiling on the cerebral vessels isolated from the brains of the mice treated with peripheral lipopolysaccharide (LPS) revealed that the cerebral vasculature responds within minutes to acute systemic inflammation by upregulating the expression of immediate early response genes, followed by activation of the nuclear factor-κB pathway. To identify the earliest responding cell type, we used fluorescence-activated cell sorting (FACS) to sort the glial and vascular cells from the brains of the mice treated with LPS at different time points, and RNA-seq was performed on microglia and cerebral endothelial cells (CECs). Bioinformatic analysis followed by further validation in all the cell types revealed that panendothelitis. i.e., the activation of CECs is the earliest event in the CNS during the inception of acute neuroinflammation. Microglial activation occurs later than that of CECs, suggesting that CECs are the most likely initial source of proinflammatory mediators, which could further initiate glial cell activation. This is then followed by the activation of apoptotic signaling in the CECs, which is known to lead to the blood–brain barrier disruption and allow peripheral cytokines to leak into the CNS, exacerbate the gliosis, and result in the vicious neuroinflammatory cascade. Together, our results model the earliest sequential events during the advancement of systemic inflammation into the CNS and facilitate to understand the interplay between the vascular and glial cells in initiating and driving acute neuroinflammation during SAE.

## Introduction

Sepsis-associated encephalopathy (SAE) is an acutely progressing brain dysfunction that accompanies systemic inflammatory response syndrome (SIRS) or sepsis-induced systemic inflammation and is characterized by the absence of direct central nervous system (CNS) infection [1]. It is the most common type of encephalopathy encountered by patients in the intensive care unit [2] and about 750,000 cases are reported annually in the United States alone [3]. It is very often associated with increased mortality and recovered patients often display chronic neurological dysfunction with cognitive deficits [1, 2].

Neuroinflammation, vascular dysfunction, and blood-brain barrier (BBB) disruption are all proposed to play important roles in the progression of SAE [4]. A widely accepted mechanism proposes that altered brain perfusion and cerebral endothelial cell (CEC) activation results in impaired BBB and subsequent cerebral dysfunction [3, 5, 6]. Microglial and astrocytic activation also occur, resulting in neuroinflammation and an increase in the CNS cytokine levels and thus causing delirium and sickness behavior [7, 8]. Vascular dysfunction and glial cell activation are the two major components of this mechanism. Previous studies suggest that the cerebral blood vessels are initially affected in the CNS during acute systemic inflammatory conditions [9] and that they mediate further cytokine dependent signaling [10]. *Nfkbia* was found alongside the vasculature near the circumventricular organs at 30–60 min after the induction of systemic inflammation [9, 11]. However, the global transcriptional profiles of inflammatory signaling during such and the underlying pathophysiology are not completely characterized. In particular, the immediate effects of systemic inflammation on the cerebral vasculature before advancing to vascular dysfunction are not fully understood yet.

The cells of cerebral vasculature include the vascular endothelial cells and pericytes surrounded by the astrocyte endfeet, which together form the BBB [12]. BBB is an anatomical gateway of the cerebrovascular system responsible for the immune privilege of the CNS, which isolates the CNS from the peripheral immune system and regulates physiological communication between them [13]. Initial effects of systemic inflammation on the cerebral vasculature include molecular changes that are anatomically nondisruptive in nature and are believed to occur earlier during the initiating phase of SAE [14]. There is little knowledge about the cell types in which such initial molecular events occur. The chronology of such molecular sequelae leading to SAE remains elusive. Furthermore, the cellular pathophysiology of the onset of SAE remains poorly understood. It is unclear whether vascular activation or microglial activation occurs initially, which has a determining role in the SAE progression. To prevent sepsis-associated BBB dysfunction and neuro-cognitive damages, it is imperative to know the immediate events and the precise order in which they occur in the CNS during the initiation of SAE, as well as the contribution and interplay of the cell types involved.

Sepsis is known to cause long-term structural changes in the forebrain, which leads to impaired performance in learning and memory tasks [15, 16]. In the present study, we thus used the forebrain, i.e., cortex including the hippocampus, which are crucial for learning, memory, and other cognitive functions, to find out if the cerebral vessels elicit rapid inflammatory responses, and to identify which cells in the CNS are the earliest responders to systemic inflammation.

## Methods

### Animals

C57BL/6 mice were obtained from Jackson Laboratories, housed, and bred in well-ventilated cages under standard laboratory conditions on 12:12 hour light–dark cycle with food and water ad libitum. Both male and female mice aged between 8–12 weeks were used unless otherwise mentioned. All animal experimental procedures were conducted in accordance with the animal care standards of the National Institute of Health and were approved by the Institutional Animal Care and Use Committee (IACUC) of the University of Tennessee Health Science Center.

### Lipopolysaccharide (LPS) treatment

Mice were randomized into experimental groups and received a single intraperitoneal injection of either endotoxin-free phosphate buffered saline (PBS) or LPS from *Escherichia coli* O55:B5(Sigma-Aldrich L2880) dissolved and diluted in endotoxin-free PBS (10 mg kg^−1^ body weight [17–20]). Mice were killed at specific time points post injection.

### Standard quantitative real-time polymerase chain reaction (RT-qPCR) on forebrain tissue samples

Total RNA was extracted using the Trizol method and cDNA synthesis was performed using the SuperScript™ IV VILO™ Master Mix with ezDNase™ Enzyme (Invitrogen, 11766050) according to the manufacturer’s protocols. All primer sequences were obtained from the primer bank [21–23] (Listed in supplementary table. 1) or from previously published and validated protocols [24, 25] (Listed in supplementary table. 2). RT-qPCR reactions were performed using 2× SsoAdvanced Universal SYBR Green Supermix (Bio-Rad, 1725271) on an Eppendorf Mastercycler realplex2.

### Microfluidic RT-qPCR on samples of cerebral vessels and sorted cells

Total RNA was extracted from cerebral vessels or sorted cells using the RNeasy Plus micro kit (Qiagen, 74034) and cDNA synthesis was performed as described above. RT-qPCR was performed as per the protocol described [24]. In brief, 1.25 μl of each cDNA sample was pre-amplified using 2.5 μl of 2× Taqman pre-amplification master mix (Applied Biosystems, 4391128) and 1.25 μl of the primer pool (200 nm final conc of the primer pool). Pre-amplification was performed using 10 min 95 °C denaturation step and 14 cycles of 15 seconds at 95 °C and 4 min at 60 °C. The reaction products were then cleaned up to remove unincorporated primers by using Exonuclease I treatment as per the protocol in the Appendix C from the Fluidigm Biomark RT-PCR user guide (Fluidigm PN 68000088 N1). Reaction products were diluted 10× in DNA resuspension buffer (Teknova, T0221). In all, 5 μl from a sample mix containing pre-amplified cDNA and amplification master mix of 20× SsoFast EvaGreen Supermix with Low ROX (Bio-Rad, 1725211) was loaded into each sample inlet of either a 48.48 or a 96.96 Dynamic Array IFC for gene expression (Fluidigm, BMK-M-48.48/BMK-M-96.96). In all, 5 μl from an assay mix containing DNA-assay loading reagent, as well as forward and reverse primers (10 μm final conc) was loaded into each detector inlet. The chip was then placed in either the IFC Controller MX for the 48.48 Dynamic array IFC or IFC controller HX for the 96.96 Dynamic array IFC for loading and mixing. After loading, the chip was processed in the BioMark RealTime PCR System (Fluidigm) using a cycling program of 60 seconds at 95 °C followed by 40 cycles of 96 °C for 5 seconds, 60 °C for 30 seconds and 72 °C for 30 seconds. After completion of the RT-qPCR run, a melting curve of amplified products was determined. RT-qPCR data were normalized using the geometric mean of three reference genes *Actb*, *Gapdh*, and *Rplp0*, analyzed using comparative C_t_ method [26] and following the MIQE (minimum information for publication of quantitative real-time PCR experiments) guidelines [27].

### RNA sequencing (RNA-seq)

(Cerebral vessels, Sorted CECs, and microglia) Isolated RNA sample quality was determined using High Sensitivity RNA Tapestation (Agilent Technologies Inc., California, USA) and concentration was measured using Qubit 2.0 RNA High Sensitivity assay (ThermoFisher, Massachusetts, USA). Libraries were then constructed following manufacturer’s instructions for SMART-Seq® v4 Ultra® Low Input RNA Kit (Takara Bio USA Inc., California, USA) followed by Nextera® XT DNA Library Prep Kit (Illumina, California, USA). Library concentration was initially measured using a Qubit 2.0 fluorometer (Life Technologies) and then diluted to 2 ng/µl before checking insert size on an Agilent 2100 and quantifying to greater accuracy by KAPA SYBR® FAST quantitative PCR (Roche, Indianapolis, USA) (library activity >2 nM). The resulting final library size was ~430 bp with an insert size of ~300 bp. Illumina® 8-nt dual-indices were used. Equimolar pooling of libraries was performed basing on QC values and sequencing was performed on an Illumina® NovaSeq 6000 (Illumina, California, USA) for the cerebral vessel samples or Illumina® HiSeq 4000 (Illumina, California, USA) for sorted CECs and microglia samples by Novogene Co. (Sacramento, CA). The read length configuration was 150 PE for 40 M PE reads per sample (20 M in each direction).

### RNA-seq data analysis

Transcript abundance from RNA-seq reads were quantified using Salmon [28], and gene-level counts were obtained using tximport [29], against the C57BL/6 J mouse genome annotation Genome Reference Consortium Mouse Build 38 patch release 6 (GRCm38.p6), obtained from the National Center for Biotechnology Information. Subsequently, raw counts were processed with DESeq2 [30] to determine differentially expressed genes. All statistical analyses were conducted using R version 4.0. Multiple hypothesis correction was done using the Benjamini–Hochberg method, and a *P* adjusted value (*p*.adj) of 0.05 was considered significant. R package ggplot2 [31] was used for plotting MA plots. Heatmaps were generated after normalization of the raw counts using DESeq2, accounting for library size and removing heteroskedasticity of the counts, and finally, the values were *z*-scored gene wise. *Z-*scores were calculated and obtained on a gene-by-gene basis by subtracting the mean and then dividing by the standard deviation. In pheatmap, *Z-*scores are computed after the hierarchical clustering, so that it only affects the visualization. R package clusterProfiler [32] was used to determine gene ontology (GO) categories that were enriched in the significant genes from each pairwise comparison. GO terms represented in the dot plots were selected from the top 10 inflammatory pathways affected in comparison of LPS 4 hr vs PBS in cerebral vessel samples, and these GO terms were used throughout the study to monitor the progression of inflammatory gene expression. The function enrichKEGG in clusterProfiler was used to find the Kyoto Encyclopedia of Genes and Genomes (KEGG) pathways that were enriched from the gene sets in each of the pairwise comparisons. All significant genes (*p*.adj < 0.05 and LFC > 1.5), which were differentially expressed were used, and a *q* value cutoff of 0.01 was applied. Signaling pathway impact analysis (SPIA) was done using the R package SPIA [33] to obtain the perturbation plots of the nuclear factor-κB (NF-κB) pathway (KEGG ID mmu04064) at each treatment time point in each of the cell types.

### Statistics

All statistical analyses (excluding RNA-seq data) were conducted using GraphPad Prism v. 8 (GraphPad Software, San Diego, CA). Data are presented as mean ± SEM. The sample size for each experiment and the statistical testing methods are reported in the figure legends.

Additional methods are included in the supplementary file.

## Results

### Cerebral vessels elicit proinflammatory responses during SAE

**Fig. 1:**
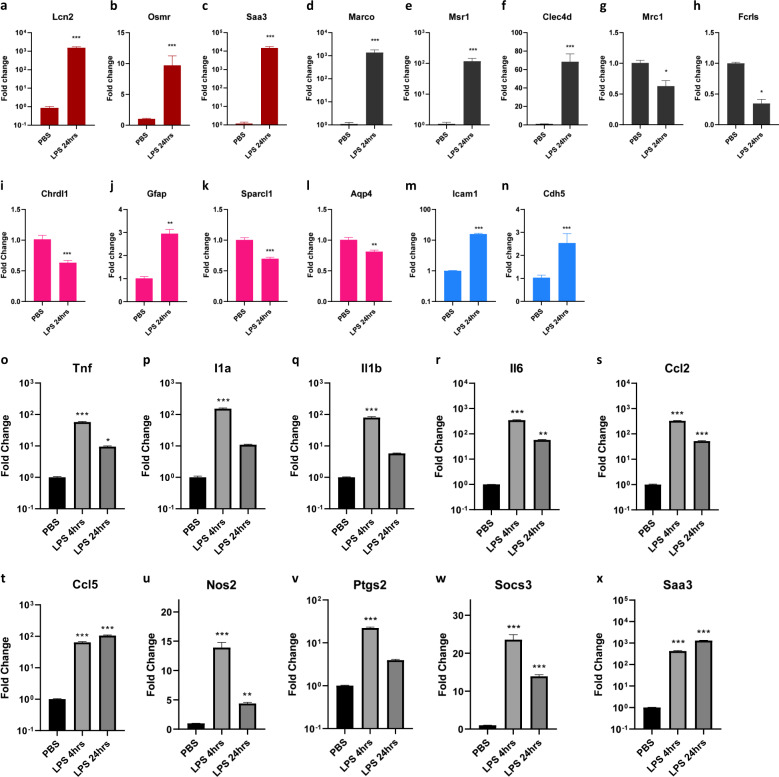
Cerebral vessels elicit proinflammatory responses during SAE. **a**–**n** mRNA fold-change levels in whole forebrain lysates from the mice injected with 10 mg/kg LPS for 24 h, **a**–**c** pan reactive genes *Lcn2, Osmr, Saa3*, **d**–**h** microglia-related genes *Marco, Msr1, Clec4d, Mrc, Fcrls*, **i**–**l** Astrocyte related genes *Chrldl1, Gfap, Sparcl1, Aqp4* (**m**, **n**). CEC related genes *Icam1* and *Cdh5*. (n = 6–8 (male and female) mice in each group, Mann–Whitney test, *p < 0.05, **p < 0.01, ***p < 0.001). **o**–**x** mRNA fold-change levels of various proinflammatory genes in cerebral vessels isolated from the brains of the mice injected with 10 mg/kg LPS for 4 h and 24 h, **o**–**r** proinflammatory cytokines *Tnf, Il1a, Il1b, Il6* **s**, **t** chemokines *Ccl2, Ccl5,* **u***–***x** inflammation-induced genes *Nos2, Ptgs2, Socs3,* and *Saa3*, (*n* = 3–4 male mice in each group, *n* = 3–4 male mice in each group, One-way ANOVA, Dunnett’s post hoc test, *p < 0.05, **p < 0.01, ***p < 0.001, asterisks indicate significance at that particular treatment time point as compared with the PBS group.

To examine the effects of systemic inflammation on the CNS, we initially looked at the gene expression in the whole forebrains isolated from the mice injected intraperitoneally with LPS. We detected significant changes in the widely reported inflammatory cytokine and chemokine gene expression (Supplementary Fig. 1a–h). We also detected changes in pan reactive genes *Lcn2, Osmr, Saa3* (Fig. 1a–c) as well as microglia related *Marco, Msr1, Clec4d, Mrc1, Fcrls* (Fig. 1d–h), astrocyte-related *Chrdl1, Gfap, Sparcl1, Aqp4* (Fig. 1i–l), and CEC-related *Icam1, Cdh5* (Fig. 1m, n). To study the effects on the cerebral vasculature, we isolated the cerebral vessels from the brains of the mice. To confirm the identity of these isolated vessels we stained them for vascular cell markers GLUT1, COL-IV, CD13, and CD31 (Supplementary Fig. 1i–k). Also, several canonical CEC marker genes were found to be selectively enriched in the isolated vessels compared with the vessel depleted brain fractions (Supplementary Fig. 1l–t). To examine if the inflammatory changes could be detected in the cerebral vasculature, we performed RT-qPCR analysis of the RNA from the cerebral vessels isolated from the mice after LPS challenge for 4 h and 24 h. Significant alteration of proinflammatory cytokine genes *Tnf, Il1a, Il1b*, and *Il6* (Fig. 1o–r), and chemokine genes *Ccl2* and *Ccl5* (Fig. 1s, t), as well as proinflammatory genes *Nos2, Ptgs2, Socs3,* and *Saa3* (Fig. 1u–x), demonstrated the effect of systemic inflammation on the cerebral vessels in the CNS.

### Acute systemic inflammation rapidly affects the cerebral vessels during the initiation of SAE

**Fig. 2:**
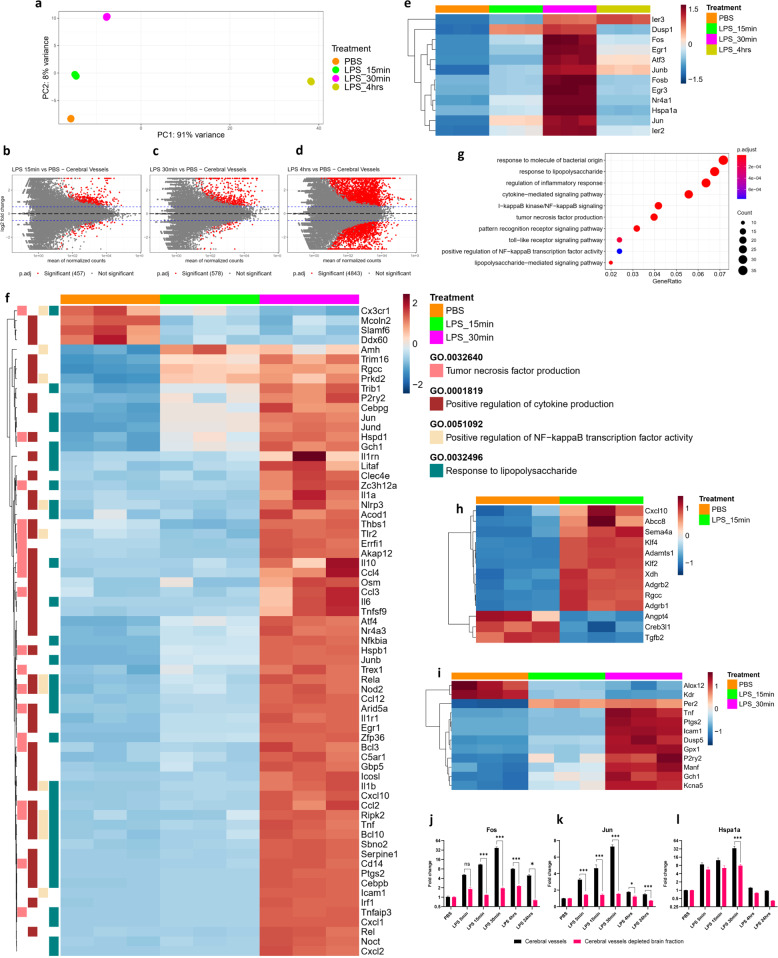
Acute systemic inflammation rapidly affects the cerebral vessels during the initiation of SAE. **a** PCA plot of the RNA-seq data from cerebral vessels isolated from the brains of mice injected with 10 mg/kg LPS for 15 min, 30 min, and 4 h or PBS. (*n* = 3 in each group, every sample in each group includes the cerebral vessels from one male and one female mouse pooled together) **b**–**d**. MA plots of the pairwise comparisons of cerebral vessels for the LPS treatment timepoints **b** 15 min, **c** 30 min, and **d** 4 h compared with PBS; log fold changes (LFCs) are plotted against the mean of normalized counts to determine the variance between two treatments in terms of gene expression. Red nodes on the graph represent statistically significant data points, i.e., *p*.adj < 0.05 and LFC > 1.5. Gray nodes are data points that are not statistically significant. Numerical values in parentheses for the significant legend indicate the number of genes that meet the prior condition. Dashed lines indicate the cutoff LFC values. e. Heatmap showing the progression of immediate early genes activation in the cerebral vessels, (significant genes (*p*.adj < 0.05 and LFC > 1.5) in LPS 15 min, 30 min, and 4 h compared with PBS). **f** Heatmap showing the progression of significant genes belonging to gene ontology (GO) terms positive regulation of NF-κB activity (GO:0051092), response to lipopolysaccharide (GO:0032496), positive regulation of cytokine production (GO:0001819), and tumor necrosis factor production (GO:0032640), in the cerebral vessels from the LPS treatment time points 15 min and 30 min (significant genes (*p*.adj < 0.05 and LFC > 1.5) in LPS 30 min compared with PBS). **g** GO enrichment dot plot showing the number of genes affected in the inflammation-related GO terms in the cerebral vessels from 30 min LPS treatment compared with PBS. **h** Heatmap showing the significant (*p*.adj < 0.05 and LFC > 1.5) genes belonging to gene ontology term negative regulation of blood vessel morphogenesis (GO:200018), in the cerebral vessels from the LPS treatment time point 15 min. i. Heatmap showing the progression of significant genes belonging to gene ontology term regulation of blood vessel diameter (GO:0097746), in the cerebral vessels from the LPS treatment time point 15 min and 30 min (significant genes (*p*.adj < 0.05 and LFC > 1.5) in LPS 30 min compared with PBS). **j**–**l** Time course of **j** *Fos*, **k** *Jun*, **l** *Hspa1a* mRNA fold changes in cerebral vessels and cerebral vessels depleted fractions, isolated from the brains of mice treated with 10 mg/kg LPS for 5 min, 15 min, 30 min, 1 h, 4 h, 24 h, and PBS, graphs depict mean ± SEM, *n* = 8 (four males and four females in each group), Two-way ANOVA with Dunnett post hoc test, *p < 0.05, **p < 0.01, ***p < 0.001.

After detecting the transcriptional changes in the isolated cerebral blood vessels during systemic inflammation, we next wanted to know how soon do the changes occur in the cerebral vasculature in response to systemic inflammation. To capture the global gene expression patterns in the cerebral vasculature following acute systemic inflammation, RNA-seq was performed on the isolated cerebral vessels from the mice at 15 min, 30 min, and 4 h after receiving LPS. The scatterplot matrix shows the expression levels of all the pairwise comparisons of the LPS treatments to PBS control (Supplementary Fig. 2a). Analysis of the RNA-seq data followed by the principal component analysis (PCA) showed a clear separation among these treatment time points (Fig. 2a). Moreover, unsupervised hierarchical clustering of samples also displayed distinct grouping according to the treatment groups (Supplementary Fig. 2b). Further analysis revealed the differential expression of 457, 558, and 4843 genes significantly (*p*.adj < 0.05 and log fold change (LFC) > 1.5) during the treatment timepoints 15 min, 30 min, and 4 h respectively compared with the baseline PBS control (Fig. 2b–d). We then looked at the progression of immediate early genes [34] (IEGs), which indicate the earliest inflammatory responses. There was a rapid induction of IEGs in the blood vessels as early as 15 min and peaking at 30 min post LPS (Fig. 2e, Supplementary Table 3a–c). We also observed the changes in the genes belonging to the GO term response to LPS (GO:0032496) at 15 min, suggesting the immediate effect of peripheral inflammation on the cerebral blood vessels (16 genes, Supplementary Fig. 2c, Supplementary Table 4). Next, we observed distinct changes at 30 min in the genes belonging to positive regulation of NF-κB transcription factor activity (GO:0051092), response to LPS (GO:0032496), positive regulation of cytokine production (GO:0001819), and tumor necrosis factor production (GO:0032640) (67 genes, Fig. 2f, Supplementary Table 5), suggesting the rapid activation of proinflammatory signaling. GO enrichment was done on the differentially expressed significant genes, and the dot plots show the progression of selected GO terms related to inflammatory signaling at each time point, further indicating the immediate induction of proinflammatory signaling in the vasculature as early as 15 min after peripheral LPS injection (Supplementary Fig. 2d; Fig. 2g and Supplementary Fig. 2e). Our results indicate that *Nfkbia* begins to increase as early as 15 min following the peripheral inflammatory insult and is also consistent with the previous reports of detecting its expression at 30 min near the vasculature in the brain [9]. Furthermore, the genes regulating the blood vessel morphology were also vastly affected at 15 min (Fig. 2h, Supplementary Table 6), and the genes regulating the blood vessel size and diameter at 30 min (Fig. 2i, Supplementary Table 7). At 4 h, the genes regulating the establishment of endothelial barrier were affected which suggest the functional effects of proinflammatory signaling on the vasculature, i.e., rapid progression towards BBB disruption (Supplementary Fig. 2f, Supplementary Table 8). The dot plot shows the changes in GO terms related to the BBB integrity, and the number of associated genes which were significantly affected at 4 h post LPS injection (Supplementary Fig. 2g). Together, these results suggest the progression of inflammatory response into the CNS by beginning with the activation of inflammatory signaling in the vasculature and simultaneously affecting the blood vessel size and diameter, continued by rapid inflammatory signaling, and finally the induction of endothelial apoptotic signaling and dysregulation of the endothelial barrier.

To further confirm the RNA-seq results and to know how earliest the peripheral inflammation induces the changes in the cerebral vasculature, we performed a similar time course study to look at the pattern of the changes in the gene expression of the blood vessels. We thus isolated the cerebral vessels from the brains of the mice which were injected with LPS and killed after 5 min, 15 min, 30 min, 1 h, 2 h, and 4 h. RT-qPCR results confirmed the rapid induction of immediate early response genes *Fos, Jun*, and *Hspa1a1* in the blood vessels (Fig. 2j–l). Of note, this early response was found enriched in the vessels compared with the remaining brain fractions after vessel depletion, suggesting the important role of vasculature-mediated inflammatory wave propagation into the CNS. We also found that the genes related to the GO terms response to LPS and positive regulation of NF-κB signaling were significantly altered during the initiation of acute neuroinflammation, which confirmed the results obtained from the RNA-seq analysis on the cerebral vessels (Supplementary Fig. 2h, i).

### Cerebrovascular cells are affected during SAE

**Fig. 3:**
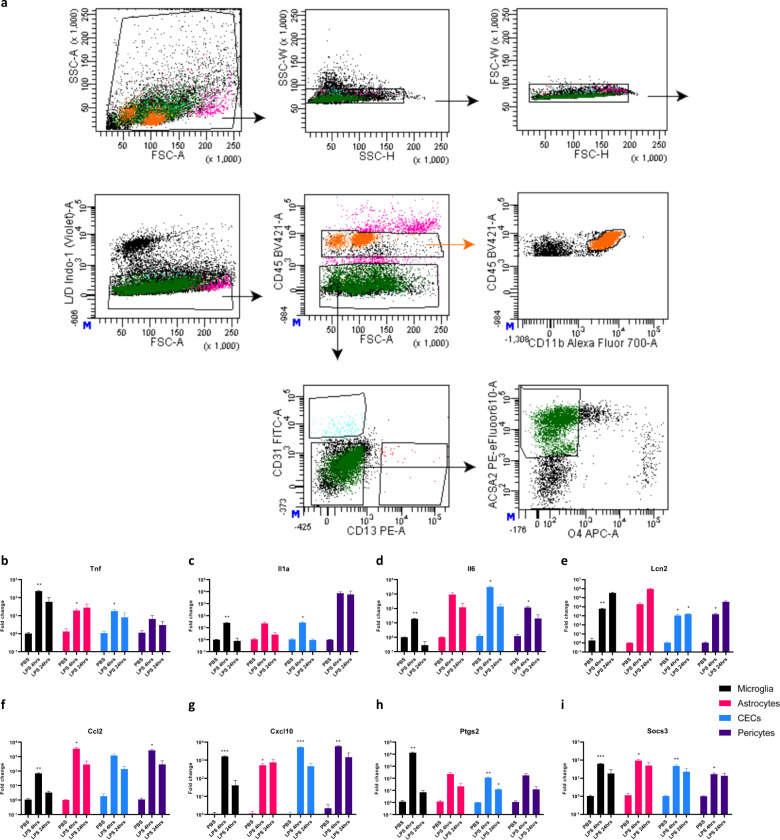
Cerebrovascular cells are affected during sepsis-induced neuroinflammation. **a** Fluorescence-activated cell sorting (FACS) strategy for the simultaneous sorting of gliovascular cells from an adult mouse brain, microglia, CECs, pericytes, astrocytes were defined and gated as CD45 low-mid CD11b+, CD45− CD13− CD31+, CD45− CD31− CD13+, and CD45− CD31− CD13− O4− ACSA2+ respectively, **b**–**i** Time course of mRNA fold change in microglia, astrocytes, CECs, and pericytes isolated from the brains of mice injected with 10 mg/kg LPS for 4 h and 24 h, **b** *Tnf*, **c** *Il1a*, **d** *Il6*, **e** *Lcn2*, **f** *Ccl2*, **g** *Cxcl10*, **h** *Ptgs2*, and **i** *Socs3*. Graphs depict mean ± SEM, *n* = 4 (females) each group, Two-way ANOVA with Dunnett post hoc test, *p < 0.05, **p < 0.01, ***p < 0.001 asterisks indicate significance at that particular treatment time point as compared with the respective PBS group in each of the cell types.

To identify which specific cell type(s) were responsible for the transcriptional changes detected in the cerebral vessels as well as to pinpoint the individual contribution of each of the predominant cell types in the CNS, we designed a flow cytometry surface staining panel and validated a gating scheme to simultaneously label and sort all the major non-neuronal gliovascular cells (CECs, microglia, astrocytes, and pericytes) from an adult mouse brain. We then injected the mice with LPS and killed them after 4 h and 24 h, immediately followed by the isolation of the brains and dissociation to yield single-cell suspension, immunolabelling, and finally sorting them using fluorescence-activated cell sorting (FACS). Microglia, CECs, pericytes, astrocytes were defined and gated as CD45 low-mid CD11b+ [35], CD45− CD13− CD31+ [36, 37], CD45− CD31− CD13+ [37], and CD45− CD31− CD13− O4− ACSA2+ [38, 39] respectively, as shown in the representative gating scheme (Fig. 3a). Only surface markers were used to avoid cell permeabilization, and dead cells were excluded to ensure respective cell type identity and sorted cell quality during the sorting. We then performed RT-qPCR on the RNA isolated from the sorted individual cell lysates. Our results revealed that not only the microglia and astrocytes responded to acute systemic inflammation, but also the cerebrovascular endothelial cells, as well as pericytes, displayed proinflammatory transcriptional changes at 4 h and 24 h post peripheral inflammatory insult (Fig. 3b–i), suggesting that the results we obtained earlier on the isolated cerebral vessels might be due to the responses in these vascular cells. To further confirm the identity of the sorted cells, we analyzed the lysates for the cell type specific marker genes using RT-qPCR, i.e., *Aif1/Iba1, Siglech, Tmem119,* *Csf1r,* and *Adora3* for microglia, *Aldh1l1, Gfap*, *S100b,* and *Aqp4* for astrocytes, *Cdh5, Ocln, Glut1*, and *Cldn5* for CECs, *Anpep*, and *Pdgfrb* for pericytes. We then found the appropriate enrichment or depletion of each cell type specific marker transcripts in the respective sorted immunolabelled target populations, further confirming the purity of the isolated cell populations (Supplementary Fig. 3a–o).

### Temporal transcriptional profiling of CECs and microglia reveal individual cell type-specific gene expression kinetics during the initiation of SAE

**Fig. 4:**
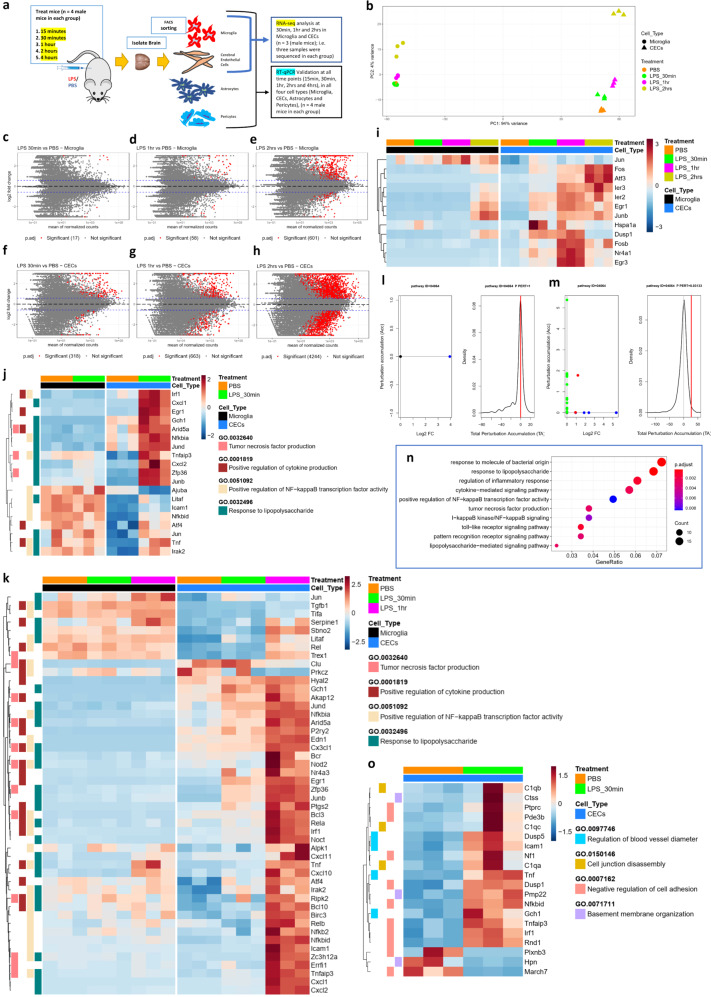
Temporal cell type-specific transcriptional profiling of CECs and microglia following systemic inflammation. **a** Scheme illustrating the experimental design—mice were injected with an intraperitoneal 10 mg/kg LPS for 15 min, 30 min, 1-hour, 2 h, and 4 h or with PBS, followed by FACS to collect the gliovascular cells (*n* = 4 (males) each group; from these samples, RNA-seq was performed on CECs and microglia at the LPS treatment timepoints 30 min, 1 h, 2 h and PBS. *n* = 3 (males) each group; RT-qPCR validation was done on microglia, CECs, astrocytes and pericytes at the LPS treatment timepoints 15 min, 30 min, 1 h, 2 h, 4 h, and PBS, *n* = 4 (males) each group. **b** PCA plot of the RNA-seq data from microglia and CECs isolated from the brains of mice injected with 10 mg/kg LPS for 30 min, 1 h, and 2 h or PBS *n* = 3 (males) each group. **c**–**h** MA plots of the pairwise comparisons in microglia between the time points **c** 30 min, **d** 1 h, and **e** 2 h compared with PBS (microglia); and in CECs for the LPS treatment timepoints **f** 30 min, **g** 1–h, and **h** 2 h compared with PBS (CECs). log fold changes (LFCs) are plotted against the mean of normalized counts to determine the variance between two treatments in terms of gene expression. Red nodes on the graph represent statistically significant data points, i.e., *p*.adj < 0.05 and LFC > 1.5. Gray nodes are data points that are not statistically significant. Numerical values in parentheses for the significant legend indicate the number of genes that meet the prior condition. Dashed lines indicate the cutoff LFC values. **i** Heatmap showing the progression of immediate early genes in the microglia and CECs, (significant genes (*p*.adj < 0.05 and LFC > 1.5) in CECs, at LPS 30 min, 1 h, and 2 h compared with PBS). **j**, **k** Heatmaps showing the progression of significant genes belonging to gene ontology terms positive regulation of NF-κB activity (GO:0051092), response to lipopolysaccharide (GO:0032496), positive regulation of cytokine production (GO:0001819) and tumor necrosis factor production (GO:0032640), in microglia and CECs from the LPS treatment time points **j** 30 min (significant genes (*p*.adj < 0.05 and LFC > 1.5) in CECs LPS 30 min compared with CECs PBS) and **k** 30 min and 1 h (significant genes (*p*.adj < 0.05 and LFC > 1.5) in CECs LPS 1 h compared with CECs PBS). **l**, **m** Perturbation plots for the NF-κB pathway (Kyoto Encyclopedia of Genes and Genomes (KEGG) ID mmu:04064) **l** in microglia from treatment time point LPS 30 min compared with PBS **m** in CECs from the treatment time point LPS 30 min compared with PBS. The perturbation of all genes in the pathway are depicted as a function of the log2 fold changes (left panel). Non differentially expressed genes are assigned 0 log2 fold-change. The null distribution of the net accumulated perturbation is also shown as a gray vertical line (right panel). The observed total accumulation (tA) with the actual data is shown as a red vertical line (right panel). **n** GO enrichment dot plot showing the number of genes affected in the top 10 enriched inflammation-related GO terms in the CECs from 30 min LPS treatment compared with PBS. **o** Heatmap showing the significant genes belonging to gene ontology terms regulation of blood vessel diameter (GO:0097746), cell junction disassembly (GO:0150146), negative regulation of cell adhesion (GO:0007162), basement membrane organization (GO:0071711) in CECs from the LPS treatment time point 30 min (significant genes (*p*.adj < 0.05 and LFC > 1.5) in CECs LPS 30 min compared with CECs PBS).

Though we were able to identify that the vascular cells are affected during systemic inflammation, it is still unclear about which cell type in the vasculature initially senses or responds to acute systemic inflammation and additionally, if this response is a result of peripheral inflammation or a result of glial cell responses in the CNS. After the initial validation of our method to sort the various cells in the CNS, we next wanted to find out which vascular cell type mediates the acute inflammatory responses detected in the cerebral blood vessels and to determine how these responses compare against that of the responses in resident myeloid cells, i.e., microglia. Such a comparative study would allow us to sequentially visualize and contrast the cell type-specific progression of inflammatory events as a function of time. Given that endothelial cells being the principal vascular cell type lining up the blood vessels, we hypothesized that CECs could be the key players in mediating the acute inflammatory responses seen in the cerebral vasculature. We thus treated the mice with LPS for 15 min, 30 min, 1 h, 2 h, and 4 h, followed by FACS to collect the gliovascular cells (Fig. 4a). RNA-seq was then performed on the RNA from CECs and microglia at the time points 30 min, 1 h, 2 h, and PBS. We chose 30 min as this was the time point when we saw a maximal initial response in the isolated cerebral vessels. The scatterplot matrices show the expression levels of all the pairwise comparisons of the LPS treatments to PBS control in microglia (Supplementary Fig. 4a) and CECs (Supplementary Fig. 4b). RNA-seq data analysis followed by the PCA analysis showed a clear separation among the cell type (PC1–94% variance) as well as the treatment (PC2–4% variance) (Fig. 4b). We further confirmed the cell type identities by comparing microglia and CECs; and found the exclusive enrichment of several cell type-specific markers in each of the respective cell types (Supplementary Fig. 4c). Further analysis to determine the effect of LPS treatment within each cell type revealed the differential expression of 17, 58, and 601 genes in microglia (Fig. 4c–e); whereas 318, 663, and 4244 genes in CECs (Fig. 4f–h) significantly (*p*.adj < 0.05 and LFC > 1.5) during the treatment timepoints 30 min, 1 h, and 2 h in each, respectively. These differentially expressed significant genes were obtained by using the Wald test in DESeq2 analysis and computing the individual pairwise comparison to the respective cell type PBS control, at each of the time points. We next observed the rapid induction of immediate early response genes exclusively in CECs but not in microglia as early as 30 min and peaking at 1 h post LPS (Fig. 4i, Supplementary Table 9a–e), suggesting the immediate activation of CECs during systemic inflammatory conditions. From each set of the significant genes from the pairwise comparisons at each time points in microglia and CECs, we closely looked at the genes related to the GO biological process terms positive regulation of NF-κB transcription factor activity (GO:0051092), response to LPS (GO:0032496), positive regulation of cytokine production (GO:0001819) and tumor necrosis factor production (GO:0032640) to monitor the progression of peripheral inflammation into the CNS. We found clear changes in expression of 19 genes belonging to the above GO terms in CECs at 30 min (Fig. 4j, Supplementary Table 10). Strikingly, there were no genes found to be altered in microglia at 30 min, indicating that there is not yet an induction of inflammatory responses in microglia at 30 min. At 1 h time point, we found the changes in 48 genes in CECs (Fig. 4k, Supplementary Table 11), whereas seven genes in microglia appear to change (Supplementary Fig. 4d, Supplementary Table 12), together suggesting that the inflammatory response occurs earlier in the CECs at 30 min followed later in microglia at 1 h in the CNS. The perturbation plots obtained from the signaling pathway impact analysis revealed the activation of the NF-κB pathway in CECs but not in microglia at 30 min (Fig. 4l, m) as evidenced by the comparison of total accumulated perturbation. But, at 1 h, the NF-κB pathway was activated in microglia as well as CECs (Supplementary figure 4e, f), followed by a further increase in the total accumulation of perturbation at 2 h post LPS in both the cell types (Supplementary figure 4g, h). At 2 h, we found 161 genes in CECs and 71 genes in microglia, suggesting the induction of inflammatory responses in microglia too alongside CECs.

GO enrichment was done on the significant genes, and the dot plots show the progression of selected GO terms related to inflammatory response at 30 min in CECs (Fig. 4n), whereas no enrichment was found in microglia plausibly because of very few significantly changed genes. However, at 1 h, whereas CECs have substantially increased differentially expressed genes in each GO term compared with the earlier 30 min, indicating the progression of inflammation, few GO terms begin to be enriched in microglia (Supplementary Fig. 4i, j). Finally, at 2 h, all the GO terms appear from CECs as well as from microglia, suggesting the induction of inflammatory responses in both cell types by this time point. However, there was still a much larger number of genes that were significantly affected in CECs compared with that in microglia at 2 h (Supplementary Fig. 4k, l). To investigate the pathways that are activated upon LPS treatment, enrichment analysis of the Kyoto Encyclopedia of Genes and Genomes (KEGG) pathways was done. This revealed the enrichment of the pathways which are primarily related to inflammatory signaling and to acute responses to various bacterial infections (as we used bacterial LPS to evoke systemic inflammatory conditions) in CECs at 30 min post LPS. Such an enrichment was not found in microglia at this time point (Supplementary Fig. 4m, n). However, at 1-hour, enrichment of inflammatory-related pathways was observed in microglia too alongside CECs (Supplementary Fig. 4o, p). In CECs, the total number of genes affected were found to be increased in each of the pathways compared with that of the enrichment at 30 min. This pattern continued in both the cell types at 2 h with an additional increase in the number of genes affected in each of the enriched inflammatory pathways, this time in both the cell types (Supplementary Fig. 4q, r).

In addition, likelihood ratio test (LRT) [30] was done by using a reduced model as the design formula in DESeq2, which identified the differentially expressed genes between the cell types at each time point by taking the baseline expression of the respective cell type PBS controls into consideration to determine the changes in gene expression patterns, and the significant genes were returned whose pattern of expression is different from that of the reference cell type. This further revealed that 90, 202, and 2078 genes were differentially expressed in CECs compared with microglia at each of the time points 30 min, 1 h, and 2 h post LPS injection, respectively (Supplementary Fig. 4s–u). Gene ontology enrichment was done on these significant genes, and the dot plots show the initiation of all selected GO terms related to inflammatory response at 30 min (Supplementary Fig. 4v), further progressing at 1 h and 2 h in CECs compared with microglia (Supplementary Fig. 4w, x).

Besides, the genes regulating the blood vessel size and diameter, cell–cell adhesion were also affected in CECs at 30 min (Fig. 4o, Supplementary Table 13). At 2 h, the genes regulating the establishment of endothelial barrier and endothelial apoptotic signaling were affected (Supplementary Fig. 4y, Supplementary Table 14) further confirming our data on the cerebral vessels. This suggests that the functional effects on the cerebral vasculature are mediated through major transcriptional changes in the CECs, which continue to progress after the initial proinflammatory signaling, leading towards BBB disruption. The dot plot shows the changes in GO terms related to the BBB integrity, and the number of associated genes which were significantly affected in CECs at 2 h post LPS injection (Supplementary Fig. 4z). Together, these results suggest the progression of inflammatory response into the CNS by beginning with panendothelitis [40, 41] i.e., the activation of inflammatory signaling in the CECs of the vasculature, followed by the activation of microglia.

### CECs are the earliest sensors in the CNS to the peripheral inflammation

**Fig. 5:**
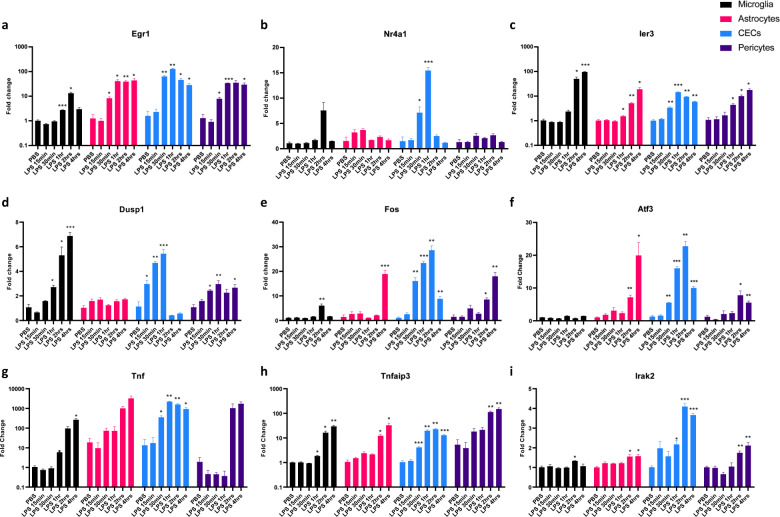
Cerebral endothelial cells are the earliest to respond to the peripheral inflammation in the CNS. **a**–**i** Time course of mRNA fold change in microglia, astrocytes, CECs, and pericytes isolated from the brains of mice injected with 10 mg/kg LPS **a** *Egr1*, **b** *Nr4a1*, **c** *Ier3*, **d** *Dusp1*, **e** *Fos*, **f** *Atf3*, **g**. *Tnf*, **h**. *Tnfaip3*, and **i**. *Irak2*. Graphs depict mean ± SEM, *n* = 4 (males) each group, two-way ANOVA with Dunnett post hoc test, *p < 0.05, **p < 0.01, ***p < 0.001, asterisks indicate significance at that particular treatment time point as compared with the respective PBS group in each of the cell types.

After identifying that the CECs in the brain respond earlier than microglia during acute systemic inflammation, we wanted to confirm our RNA-seq data and also further identify the role of astrocytes as well as pericytes to get a simultaneous global picture of any contribution from these other two cell types covering the cerebral vessels. RT-qPCR for the gene expression of the FACS-sorted samples collected earlier confirmed the initial activation of early response genes *Egr1, Nr4a1, Ier3, Dusp1, Fos, Atf3* (Fig. 5a–f) and *Egr3, Jun, Junb, Fosb, Ier2, Hspa1a1* (Supplementary Fig. 5f–k) in CECs compared with the other cell types microglia, astrocytes, and pericytes. Of note, the genes *Tnf, Tnfaip3*, and *Irak2* in the NF-κB pathway also displayed the earliest changes in CECs compared with the rest of the cell types (Fig. 5g–i). This further validated our findings from the earlier RNA-seq. In addition, we did not detect CD45 high macrophage infiltration in the CNS up to 4 h after peripheral LPS insult (Supplementary Fig. 5a–e), suggesting that the earlier changes to BBB were nondisruptive and its disruption followed by leakage is a later event to that of the initial CEC activation. Together, these results suggest that the CECs are activated and contribute to inflammatory signaling initially in the CNS, well before the functional effects on the BBB happen.

## Discussion

We report that systemic inflammation begins to exert its effects on the cerebral vasculature as early as 5 minutes. Furthermore, we observed such activation only in the CECs, suggesting their role in the propagation of peripheral inflammatory responses into the CNS. CECs display the earliest changes in the transcription of proinflammatory mediators and cytokines in the CNS and are most likely the cells primarily responsible for the initiation of the acute neuroinflammatory cascade during a peripheral inflammatory insult. To our knowledge, ours is the first in vivo study providing comprehensive transcriptional insights into the immediate effects on the cerebral vasculature as well as on the vascular and glial cells in the CNS during acute systemic inflammation.

IEGs have an essential role in the acute immune responses and are transcribed within minutes of cell-extrinsic signals without the need for de-novo protein synthesis [34, 42]. Our results show the prompt upregulation of IEGs as well as changes in the genes related to blood vessel morphology, activation of the NF-κB pathway, and *Tnf* production in the isolated cerebral vessels, all indicating the immediate and rapid effects of systemic inflammation on the cerebral vasculature.

Neuroinflammation resulting from glial cell activation is a characteristic phenomenon in acutely progressing CNS conditions like SAE, which further leads to delirium and sickness behavior [4, 43, 44]. Microglial activation causes reactive astrogliosis, which progresses to widespread neuroinflammation [24]. Microglia, the resident myeloid cells in the CNS have toll-like receptors to sense pathogen-associated molecular patterns [45], but there is an absence of a direct infection in the CNS during SAE [1]. The BBB is reported to remain anatomically intact until 4 h of the initiation of the peripheral inflammation [46], which does not allow the passage of circulating LPS and peripheral cytokines into the CNS until then [47], excluding the circumventricular organs (CVOs), which lack BBB. However, even in the CVOs, the initial changes are detected near the blood vessels [9, 48–50]. A recent study revealed the effect of microglial activation on BBB integrity during neuroinflammation [51]. The precise mechanism of the initial microglial activation and the induction of neuroinflammation in-vivo are still elusive. Thus, it is important to know the sequence of molecular events happening during the initial inflammatory progression into the CNS, the identity of the cell type/s initially affected, and the inflammatory signaling mechanism responsible for initiating microglial activation, which results in further damage of BBB alongside widespread neuroinflammation.

We found that not only microglia and astrocytes but also the cells of the cerebrovascular system, i.e., CECs and pericytes respond to peripheral inflammation. However, it is important to know if such an effect on the CECs is owing to the initiation of neuroinflammation induced by microglial activation or whether it is an independent effect of peripheral inflammation, which could further lead to neuroinflammation. From our results, it can be concluded that the cerebral vasculature senses the peripheral inflammatory signals and responds by the rapid induction of IEG expression, specifically in the CECs. We also found that the CECs display rapid changes in the genes regulating vascular tone following systemic inflammation, together explaining that these changes might contribute to the microcirculatory dysfunction at cellular levels [52] seen in the cerebral blood vessels. Simultaneously, inflammatory signaling is rapidly induced by activation of the NF-κB pathway and upregulation of *Tnf* production earlier in CECs followed by microglia. Notably, our results demonstrate that the microglia are activated later than that of CECs indicating that the CECs are the earliest sensors of peripheral inflammation in the CNS, and initial triggering of microglial activation might be owing to the proinflammatory factors secreted by the activated CECs. Following the initial CEC activation and inflammatory signaling, microglial activation occurs, leading to a further increase in the expression of proinflammatory cytokines. Finally, the activation of endothelial cell apoptotic signaling and alteration of BBB occur, which together lead to apoptotic cascade in CECs, affecting the barrier integrity. Consequently, forebrain regions of the cortex and hippocampus were found to have higher barrier disruption during systemic inflammation [53]. Also, heparan fragments released from the degrading endothelium glycocalyx of the BBB during sepsis were found to sequester brain-derived neurotrophic factor and impair hippocampal long-term potentiation [54, 55] explaining the resulting cognitive impairment. Taken together, our results model the earliest sequential events during the advancement of systemic inflammation into the CNS, clearly demonstrate that the cerebral vasculature initially senses the peripheral inflammatory responses as they propagate into the CNS and that the CECs of the cerebral vasculature are the earliest responders, which rapidly initiate proinflammatory signaling in the brain during acute systemic inflammation.

However, several key questions warrant future investigation as to whether CEC activation leads to reactive astrogliosis prior to microgliosis, and which factors released by CECs can cause microglial or astrocytic activation. Astrocytes require *Tnfr1* for the inflammatory response [56] and are activated to the neurotoxic A1 phenotype by TNF [24]. As our results show that the CECs upregulate *Tnf* earlier than microglia, it could possibly lead to the activation of reactive astrocytes surrounding the cerebral vessels, as the astrocytic endfeet wrap around the blood vessels [57]. Nonetheless, whether the CEC activation directly leads to microglial activation or progresses via an intermediate initial astrogliosis followed by microgliosis, and then by the continuation of such a vicious cycle remains to be answered.

Systemic inflammatory factors affecting the brain are relevant in acutely progressing CNS disorders like encephalopathies and are also involved in the development of chronic neurodegenerative conditions like Alzheimer’s disease [58, 59]. The ongoing pandemic of COVID-19 caused by SARS-CoV-2 also leads to acute necrotizing hemorrhagic encephalopathy [60]. Our results provide insight into the mechanism of progression of peripheral inflammatory responses into the CNS as well as a glimpse into the timing for the initiation of microglial activation, which leads to neuroinflammation. These findings as such are applicable to peripheral inflammation-induced neurological complications wherein there is no direct CNS infection [61] but which primarily develop and progress due to the prevailing SIRS [60, 62]. Our finding that the peripheral inflammatory factors initially affecting the CECs could be more relevant in the context that CEC function can be modulated by systemically administered drugs to block the progression of peripheral inflammation into the CNS. Modulation of CEC activation earlier during the course of SIRS may thus represent a newer therapeutic target to possibly inhibit inflammatory signaling in CECs to halt neuroinflammation and BBB disruption during SAE. Furthermore, therapeutic targeting of CECs may not require the drugs to pass BBB and as such overcomes a major hurdle in CNS drug delivery.

In conclusion, this study facilitates filling our knowledge gap between peripheral inflammation and microglia-mediated neuroinflammation by identifying CECs as the early initiators, and microglia as the later drivers for acute neuroinflammatory cascade.

## Supplementary information

Supplementary methods and Supplementary tables

Supplementary figures 1 through 5

KEGG pathway enrichment results in CECs at 30 minutes post LPS

KEGG pathway enrichment results in microglia at 30 minutes post LPS

KEGG pathway enrichment results in CECs at 1 hour post LPS

KEGG pathway enrichment results in microglia at 1 hour post LPS

KEGG pathway enrichment results in CECs at 2 hours post LPS

KEGG pathway enrichment results in microglia at 2 hours post LPS

## Data Availability

Raw and processed RNA-seq data of the cerebral vessels and the sorted cells are available at the Gene Expression Omnibus under the accessions GSE155516 and GSE155517.

## Code availability

Code used in the study is available at GitHub and can be accessed from the following links. The code for cerebral vessel RNA-seq analysis is deposited at https://github.com/maheshchandrakodali/Cerebral-Vessels-Aug-2020-R-Code and for microglia and CECs RNA-seq analysis is deposited at https://github.com/maheshchandrakodali/Sorted-microglia-CECs-Aug-2020-R-Code.
