# Supplementary material for: Temporal unsnarling of brain’s acute neuroinflammatory transcriptional profiles reveals panendothelitis as the earliest event preceding microgliosis
Source: Mol Psychiatry. 2020 Dec 8;26(8):3905–19. doi: 10.1038/s41380-020-00955-5 (PMC7722246; doi:10.1038/s41380-020-00955-5)
Supplement: Supplementary file 1 — Supplementary methods and Supplementary tables [file 41380_2020_955_MOESM1_ESM.pdf]

Supplementary methods:

Isolation of cerebral vessels: Cerebral vessels were isolated from the mice as described [1, 2] with slight modifications. Briefly, mice were sacrificed, followed by intracardiac perfusion with ice-cold PBS and the brains were isolated rapidly on ice. Meninges were carefully removed, and the forebrains were dissected and resuspended in 20 ml of ice-cold HBSS with 10 mM HEPES, followed by homogenization in a Dounce homogenizer (placed on ice), by performing 20 strokes at a steady pace initially without twisting and followed by five more strokes with twisting. Samples were then pelleted at 2000 g for 10 minutes in a prechilled centrifuge at 4°C. Supernatant was discarded followed by the addition of 20 ml of 18% dextran in HBSS with 10 mM HEPES. Pellet was then resuspended followed by spinning at 2800 g for 30 minutes at 4^0C. Supernatant was discarded, and the pellet was finally resuspended in 5 ml of 1% BSA in HBSS, followed by filtration through a 20 µm Nylon mesh. The retained fraction on the top of the mesh was then carefully resuspended in 50 ml of HBSS containing 1% BSA and pelleted at 2000 g for 10 minutes to yield the cerebral vessels.

Brain single cell suspension: Single cell suspension of the brain was prepared by following the protocol described [3]. Briefly, dissected whole forebrains free of meninges were cut into small pieces, followed by resuspension in 2.5 ml of HBSS containing 50 units of activated papain (Worthington, LK003172) and 250 units of activated DNase (Worthington, LK003178), and incubated at 37°C for 30 minutes. Prechilled HBSS with 2 mM EDTA and 2% FBS was added to halt the digestion, and the cells were pelleted at 300 g for 10 minutes at 4°C. The cells were then gently triturated with a glass Pasteur pipette, followed by passing through a 70 µm filter (Miltenyi, 13098462). The cells were then pelleted at 300 g for 10 minutes at 4°C, and resuspended in 12 ml of 22% isotonic 4°C Percoll plus (Millipore Sigma, E0414-250ML) in 1×

HBSS and centrifuged with low break at 560 g at 4°C for 20 minutes. The top myelin debris was then carefully discarded, and the cells were washed with HBSS containing 2% FBS, and then finally resuspended in HBSS and stained with Live/Dead Fixable Blue Dead Cell stain (Thermo Fisher, L23105) and proceeded to immunolabelling in 200 ml of HBSS containing 2% FBS and 1:100 diluted mouse CD16-CD32 Pure MAB 2.4G2 Fc Block (BD Biosciences, 553141) for 10 minutes.

Immunolabelling: The cells were stained by addition of the following antibodies. 1:1000 BV421 Rat Anti-Mouse CD45 Clone 30-F11 (BD Biosciences, 563890), 1:200 PE Rat Anti-Mouse CD13 Clone R3-242 (BD Biosciences, 558745), 1:50 CD31 Rat Anti-Mouse FITC clone MEC 13.3 (BD Biosciences, 553372), 1:200 Anti-O4-APC clone REA 576 (Miltenyi, 130-119-897), 1:1000 Alexa Fluor® 700 Rat anti-CD11b (BD Biosciences, 557960), 1:200 Anti-ACSA-2-PE-Vio615 (Miltenyi, 130-116-146) or 1:200 Anti-ACSA-2-APC (Miltenyi, 130-116-245; only used in the initial sorting experiment, where anti-O4 was not included). The stained cells were then incubated on ice in the dark for 10 minutes, and then washed three times with HBSS containing 2% FBS. Finally, the cells were resuspended in HBSS containing 2% FBS, 1:100 RNase free DNase (Qiagen, 79254) and 1:500 RNasein plus RNase inhibitor (Promega, N2611).

Fluorescence-activated cell sorting (FACS): Cell sorting was performed using a BD Aria II cell sorter using the 100-micron nozzle. Cells were sorted at 4°C and collected directly into Buffer RLT-Plus (Qiagen, 1053393) containing 1:100 β-mercaptoethanol (Millipore Sigma, 444203-250ML). Appropriate single colored controls and fluorescence minus one controls (FMOs) were included and the sorting gates were setup accordingly.

Immunohistochemistry on the isolated cerebral vessels: Isolated cerebral vessels were incubated in 4% formaldehyde for 20 minutes and then washed with PBS and resuspended in 400 ml of PBS. 50 ml of this suspension was plated on Superfrost microscope slides (Fisher Scientific, 12-550-123) and air dried for 30 minutes. The mounted vessel fractions were then washed two times with PBS, followed by permeabilization and blocking with 5% BSA and 1% NP-40 in PBS for 1 hour, stained with primary antibodies 1:100 anti-rabbit GLUT1 (PA1-46152, Invitrogen) or 1:100 anti-mouse GLUT1 (ab40084, Abcam), anti-rabbit 1:100 COL-IV (MN120-6586, Novus), 1:100 anti-mouse CD13 (M101-3, MBL International), 1:50 anti-mouse CD31 (BD Biosciences, 553372) and corresponding secondary antibodies, and imaged using Olympus F1000 confocal fluorescence microscope.

Supplementary table 1:

Gene	Forward	Reverse	Primer Bank ID
Ccl2	TTAAAAACCTGGATCGGAACCAA	GCATTAGCTTCAGATTTACGGGT	6755430a1
Cd14	CTCTGTCCTTAAAGCGGCTTAC	GTTGCGGAGGTTCAAGATGTT	6753332a1
Hras1	TTTGTGGACGAGTATGATCCCA	TGCTCCCTGTACTGATGGATG	194363765c1
Il10	CTTACTGACTGGCATGAGGATCA	GCAGCTCTAGGAGCATGTGG	291575143c1
Il12a	CAATCACGCTACCTCCTCTTTT	CAGCAGTGCAGGAATAATGTTTC	226874944c1
Irak1	CCACCCTGGGTTATGTGCC	GAGGATGTGAACGAGGTCAGC	21907906a1
Hmgb1	GGCGAGCATCCTGGCTTATC	GGCTGCTTGTCATCTGCTG	6754208a1
Ly86 (Md1)	CTGCCCTCCTTGTGTGGATTC	TGGAACACTGGTCAATGGAAAG	6754588a1
Ly96 (Md2)	CGCTGCTTTCTCCCATATTGA	CCTCAGTCTTATGCAGGGTTCA	8393737a1
Nfkbia (Ikba)	TGAAGGACGAGGAGTACGAGC	TTCGTGGATGATTGCCAAGTG	6754840a1
Pglyrp1	GCCATCCGAGTGCTCTAGC	CTTGTGGTAATGCTGCACATTG	6679293a1
Ptgs2	TTCAACACACTCTATCACTGGC	AGAAGCGTTTGCGGTACTCAT	31127110a1
Rela	AGGCTTCTGGGCCTTATGTG	TGCTTCTCTCGCCAGGAATAC	6677709a1
Ripk2	ATCCCGTACCACAAGCTCG	GGATGTGTAGGTGCTTCACTG	20336736a1
Tlr2	GCAAACGCTGTTCTGCTCAG	AGGCGTCTCCCTCTATTGTATT	31981333a1
Tlr4	ATGGCATGGCTTACACCACC	GAGGCCAATTTTGTCTCCACA	10946594a1
Tlr6	TGAGCCAAGACAGAAAACCCA	GGGACATGAGTAAGGTTCCTGTT	6755815a1
Tnfrsf1a	CCGGGAGAAGAGGGATAGCTT	TCGGACAGTCACTCACCAAGT	31560799a1
Ticam1	AACCTCCACATCCCCTGTTTT	GCCCTGGCATGGATAACCA	23272109a1
Casp8	TGCTTGGACTACATCCCACAC	TGCAGTCTAGGAAGTTGACCA	33859520a1
Chuk	GGTTTCGGGAACGTCAGTCTG	GCACCATCGCTCTCTGTTTTT	242332489c1
Fadd	GCGCCGACACGATCTACTG	TTACCCGCTCACTCAGACTTC	6753812a1
Agfg1	CCCAGACTACAGGTGGAAGTG	CTTTACCCGTGGTCCCAAAAC	26340146a1
Ikbkb	ACAGCCAGGAGATGGTACG	CAGGGTGACTGAGTCGAGAC	33469101a1
Il1r1	GTGCTACTGGGGCTCATTTGT	GGAGTAAGAGGACACTTGCGAAT	6680417a1
Irak2	GGAAGCCGGTTCCTGAGAG	GGCCGGACTTTCTCCTGTTC	26329399a1
Irf3	GAGAGCCGAACGAGGTTCAG	CTTCCAGGTTGACACGTCCG	8393627a1
Map3k7	CGGATGAGCCGTTACAGTATC	ACTCCAAGCGTTTAATAGTGTCG	27881429a1
Nfkb1	ATGGCAGACGATGATCCCTAC	TGTTGACAGTGGTATTTCTGGTG	30047197a1
Nfkb2	TGGCATCCCCGAATATGATGA	TGACAGTAGGATAGGTCTTCCG	293651547c1
Nfkbib	GCGGATGCCGATGAATGGT	TGACGTAGCCAAAGACTAAGGG	24111253a1
Nfkbil1	CCCTGATGCTTACACGGACTT	CAGCCCAGAATCTGCCCAG	6754844a1
Nfrkb	GAAGGGCGTGTTTGACAAGGA	GCATCCCGAACAAGAGACAGAAT	27370138a1
Ppara	AGAGCCCCATCTGTCCTCTC	ACTGGTAGTCTGCAAAACCAAA	31543500a1
Rel	AGAGGGGAATGCGGTTTAGAT	TTCTGGTCCAAATTCTGCTTCAT	6677707a1
Tnfaip3	GAACAGCGATCAGGCCAGG	GGACAGTTGGGTGTCTCACATT	31543880a1
Tradd	GGCAGTGCATACCTGTTTTTG	AACCGCAACTGGACGATGAG	121949761c1
Ube2n	GCTGGCAGAACCAGTTCCT	TCCCTCAAAGGGGGAATCCTG	18017605a1
Ube2v1	GGACCTCCACGAACAATCTATG	GTACTTAGGCCCACACTCTATCT	12848504a1

Clec4d	ACCCGACATCCCCAACTGAT	CTCTCGTCCAGCGTAAAAAGT	6754728a1
Mrc1	CTCTGTTCAGCTATTGGACGC	CGGAATTTCTGGGATTCAGCTTC	6678932a1
Aif1	ATCAACAAGCAATTCCTCGATGA	CAGCATTCGCTTCAAGGACATA	9506379a1
Chrdl1	AACCTCCAAGCCAAAACTTTGA	CCAGTGCTACTTTTCTGGTTGTC	15341247a1
Sparcl1	GGCAATCCCGACAAGTACAAG	TGGTTTTCTATGTCTGCTGTAGC	31982800a1
Aqp4	CTTTCTGGAAGGCAGTCTCAG	CCACACCGAGCAAAACAAAGAT	33563244a1
Cdh5	CACTGCTTTGGGAGCCTTC	GGGGCAGCGATTCATTTTTCT	6753378a1
Nos2	GTTCTCAGCCCAACAATACAAGA	GTGGACGGGTCGATGTCAC	6754872a1
Ccl5	GCTGCTTTGCCTACCTCTCC	TCGAGTGACAAACACGACTGC	7305461a1
Ptgs2	TTCAACACACTCTATCACTGGC	AGAAGCGTTTGCGGTACTCAT	31127110a1
Socs3	ATGGTCACCCACAGCAAGTTT	TCCAGTAGAATCCGCTCTCCT	6671758a1
Pdgfrb	AGGAGTGATACCAGCTTTAGTCC	CCGAGCAGGTCAGAACAAAGG	226342981c1
S100b	TGGTTGCCCTCATTGATGTCT	CCCATCCCCATCTTCGTCC	6677839a1
Csf1r	TGTCATCGAGCCTAGTGGC	CGGGAGATTCAGGGTCCAAG	6681045a1
Siglech	GCTTGTGAACGGGGAACAC	CCCAGGGATATGAGGGCAG	26337567a1
Fos	CGGGTTTCAACGCCGACTA	TTGGCACTAGAGACGGACAGA	6753894a1
Jun	CCTTCTACGACGATGCCCTC	GGTTCAAGGTCATGCTCTGTTT	6754402a1
Hspa1a	TGGTGCAGTCCGACATGAAG	GCTGAGAGTCGTTGAAGTAGGC	387211a1
Cdh5	CACTGCTTTGGGAGCCTTC	GGGGCAGCGATTCATTTTTCT	6753378a1
Ocln	TTGAAAGTCCACCTCCTTACAGA	CCGGATAAAAAGAGTACGCTGG	6679162a1
Slc2a1	CAGTTCGGCTATAACACTGGTG	GCCCCCGACAGAGAAGATG	22094111a1
Cldn5	GCAAGGTGTATGAATCTGTGCT	GTCAAGGTAACAAAGAGTGCCA	31980735a1
Anpep	ACGCTCAGGAGAAGAATAGGAA	CTTAGGCAAGCGATACTGGTTC	225637486c2
Egr1	TCGGCTCCTTTCCTCACTCA	CTCATAGGGTTGTTCGCTCGG	6681285a1
Nr4a1	TTGAGTTCGGCAAGCCTACC	GTGTACCCGTCCATGAAGGTG	6754216a1
Ier3	CAGCCGAAGGGTGCTCTAC	AGCCATCAAAATCTGGCAGAAG	118130957c1
Dusp1	GTTGTTGGATTGTCGCTCCTT	TTGGGCACGATATGCTCCAG	7305423a1
Atf3	GAGGATTTTGCTAACCTGACACC	TTGACGGTAACTGACTCCAGC	31542154a1
Egr3	CCGGTGACCATGAGCAGTTT	TAATGGGCTACCGAGTCGCT	9055212a1
Fosb	TTTTCCCGGAGACTACGACTC	GTGATTGCGGTGACCGTTG	6679827a1
Junb	TCACGACGACTCTTACGCAG	CCTTGAGACCCCGATAGGGA	6680512a1
Ier2	TGACTCTGTCGGTATGGAAGAT	ACCTTGGCTGAGAGGTAGACC	31542990a1
Rplp0	AGATTCGGGATATGCTGTTGGC	TCGGGTCCTAGACCAGTGTTC	6671569a1
Actb	GGCTGTATTCCCCTCCATCG	CCAGTTGGTAACAATGCCATGT	6671509a1

Supplementary table 2:

Gene	Forward	Reverse
Lcn2	CCAGTTCGCCATGGTATTTT	CACACTCACCACCCATTCAG
Osmr	GTGAAGGACCCAAAGCATGT	GCCTAATACCTGGTGCGTGT
Saa3	GGGTCTAGAGACATGTGGCG	TCTGGCATCGCTGATGACTT
Marco	TTCTGTCGCATGCTCGGTTA	CAGATGTTCCCAGAGCCACC
Msr1	CCAGCAATGACAAAAGAGATGACA	CTGAAGGGAGGGGCCATTTT
Fcrls	GTCGCTGGGGCACTGTATGT	GCACAGGCAGAGCTTCATCAA
Aldh1l1	GCAGGTACTTCTGGGTTGCT	GGAAGGCACCCAAGGTCAAA
Gfap	AGAAAGGTTGAATCGCTGGA	CGGCGATAGTCGTTAGCTTC
Icam1	CACGTGCTGTATGGTCCTCG	TAGGAGATGGGTTCCCCCAG
Tmem119	GTGTCTAACAGGCCCCAGAA	AGCCACGTGGTATCAAGGAG
Tnf	TGTGCTCAGAGCTTTCAACAA	CTTGATGGTGGTGCATGAGA
Il1a	CGCTTGAGTCGGCAAAGAAAT	CTTCCCGTTGCTTGACGTTG
Il1b	TGCCACCTTTTGACAGTGATG	TGATGTGCTGCTGCGAGATT
Il6	TAGTCCTTCCTACCCCAATTTCC	TTGGTCCTTAGCCACTCCTTC
Cxcl10	CCCACGTGTTGAGATCATTG	CACTGGGTAAAGGGGAGTGA
Adora3	TTGCTGGCCATTGCTGTAGA	GAGTGGTAACCGTTCTATATCTGAC
Gapdh	AAGAGGGATGCTGCCCTTAC	TACGGCCAAATCCGTTCACA

Supplementary table 3a: Related to figure. 2e (Significant genes in LPS 15min compared to PBS)

	symbol	gene	baseMean	log2FoldChange	lfcSE	stat	pvalue	padj
1	Ier3	ENSMUSG00000003541	9161.92196	2.045728334	0.042339521	34.61844367	1.33E-262	1.54E-259
2	Fosb	ENSMUSG00000003545	1496.904056	2.983667967	0.110027545	21.8460566	8.47E-106	5.86E-103
3	Fos	ENSMUSG00000021250	3694.458089	3.723838654	0.074136615	42.40601815	0	0
4	Nr4a1	ENSMUSG00000023034	9818.768561	3.161392804	0.041494666	62.21023243	0	0
5	Dusp1	ENSMUSG00000024190	12240.1039	3.360882939	0.035206069	78.98873657	0	0
6	Atf3	ENSMUSG00000026628	3581.233638	3.454395236	0.087102744	33.00005376	8.11E-239	8.85E-236
7	Egr3	ENSMUSG00000033730	2850.242127	2.225509686	0.07258448	22.67026886	8.81E-114	6.52E-111
8	Egr1	ENSMUSG00000038418	11656.38061	2.837737107	0.040269	56.0663819	0	0
9	Jun	ENSMUSG00000052684	10484.16994	2.620189009	0.038193465	53.41722801	0	0
10	Junb	ENSMUSG00000052837	15095.43776	3.185924773	0.041203035	63.24594288	0	0
11	Ier2	ENSMUSG00000053560	8229.486774	2.325638553	0.043227307	40.38277394	0	0
12	Hspa1a	ENSMUSG00000091971	12366.3025	4.16665706	0.061856728	57.98329758	0	0

Supplementary table 3b: Related to figure. 2e (Significant genes in LPS 30min compared to PBS)

	symbol	Gene	baseMean	log2FoldChange	lfcSE	stat	pvalue	padj
1	Ier3	ENSMUSG00000003541	9161.92196	3.977240173	0.041130065	82.59749062	0	0
2	Fosb	ENSMUSG00000003545	1496.904056	6.194236447	0.105610276	53.15994502	0	0
3	Fos	ENSMUSG00000021250	3694.458089	5.588559737	0.073073963	68.54096228	0	0
4	Nr4a1	ENSMUSG00000023034	9818.768561	4.857440852	0.040934774	104.4940626	0	0
5	Dusp1	ENSMUSG00000024190	12240.1039	3.59612818	0.035196888	85.69303503	0	0
6	Atf3	ENSMUSG00000026628	3581.233638	6.499963503	0.084438639	70.10965051	0	0
7	Egr3	ENSMUSG00000033730	2850.242127	5.028212227	0.06986756	63.66634564	0	0
8	Egr1	ENSMUSG00000038418	11656.38061	5.127535347	0.039328259	115.6302227	0	0
9	Jun	ENSMUSG00000052684	10484.16994	3.415445587	0.037984906	74.64663962	0	0
10	Junb	ENSMUSG00000052837	15095.43776	4.779297401	0.040769667	103.0005316	0	0
11	Ier2	ENSMUSG00000053560	8229.486774	3.462289411	0.042806879	67.33238898	0	0
12	Hspa1a	ENSMUSG00000091971	12366.3025	6.501814795	0.061400419	96.44583708	0	0

Supplementary table 3c: Related to figure. 2e (Significant genes in LPS 4hrs compared to PBS)

	symbol	Gene	baseMean	log2FoldChange	lfcSE	stat	pvalue	padj
1	Ier3	ENSMUSG00000003541	9161.92196	4.089584457	0.041396173	84.78040869	0	0
2	Fosb	ENSMUSG00000003545	1496.904056	2.863883006	0.11503594	19.8536476	1.02E-87	2.90E-86
3	Fos	ENSMUSG00000021250	3694.458089	3.906971015	0.075131133	44.28218857	0	0
4	Nr4a1	ENSMUSG00000023034	9818.768561	2.202735335	0.043710279	37.12479898	1.12E-301	7.59E-300
5	Dusp1	ENSMUSG00000024190	12240.1039	1.904789172	0.036990126	35.81466998	6.53E-281	4.15E-279
6	Atf3	ENSMUSG00000026628	3581.233638	5.400087982	0.085523149	56.36003864	0	0
7	Egr3	ENSMUSG00000033730	2850.242127	2.166373387	0.075558879	20.99519471	7.26E-98	2.20E-96
8	Egr1	ENSMUSG00000038418	11656.38061	3.745870689	0.04026618	78.62356748	0	0
9	Jun	ENSMUSG00000052684	10484.16994	1.711280196	0.039719066	28.48204416	1.96E-178	8.67E-177
10	Junb	ENSMUSG00000052837	15095.43776	4.091382233	0.04120239	85.22277939	0	0
11	Ier2	ENSMUSG00000053560	8229.486774	2.220488637	0.044108855	37.19182061	9.25E-303	6.32E-301
12	Hspa1a	ENSMUSG00000091971	12366.3025	1.210243613	0.069356382	9.087031311	1.02E-19	1.31E-18

Supplementary table 4: Related to supplementary figure. 2c

	symbol	gene	baseMean	log2FoldChange	lfcSE	stat	pvalue	padj
1	Tnfaip3	ENSMUSG00000019850	9466.877995	1.432211715	0.056321332	15.13124206	1.01E-51	4.02E-49
2	Nfkbia	ENSMUSG00000021025	24691.37012	1.236751616	0.03187299	20.60527154	2.46E-94	1.55E-91
3	Tnf	ENSMUSG00000024401	274.3510962	4.285165149	0.482703364	7.675863536	1.64E-14	2.38E-12
4	Il1b	ENSMUSG00000027398	830.013287	2.057303438	0.248011252	5.956598438	2.58E-09	2.55E-07
5	Ppbp	ENSMUSG00000029372	120.4146146	-1.629218759	0.184677288	-5.681363261	1.34E-08	1.25E-06
6	Cxcl1	ENSMUSG00000029380	28342.61911	5.802165293	0.139105469	37.54104939	0	0
7	Ptgs2	ENSMUSG00000032487	32813.74795	1.234618234	0.044135262	14.83209127	9.09E-50	3.49E-47
8	Cxcl10	ENSMUSG00000034855	51363.8536	1.080808763	0.142671141	3.510231704	0.000447716	0.022690504
9	Ccl12	ENSMUSG00000035352	349.1488707	2.00887161	0.264832184	5.395385057	6.84E-08	6.03E-06
10	Gch1	ENSMUSG00000037580	2429.995259	0.799302317	0.06469625	3.389722217	0.000699635	0.033655518
11	Cnr1	ENSMUSG00000044288	278.7260238	1.071906948	0.147879605	3.326401551	0.000879751	0.041266687
12	Zfp36	ENSMUSG00000044786	18255.99707	3.341359257	0.036813804	75.00880081	0	0
13	Jun	ENSMUSG00000052684	10484.16994	2.620189009	0.038193465	53.41722801	0	0
14	Junb	ENSMUSG00000052837	15095.43776	3.185924773	0.041203035	63.24594288	0	0
15	Cxcl2	ENSMUSG00000058427	8389.878604	4.944605366	0.287604769	15.17570579	5.12E-52	2.08E-49
16	Jund	ENSMUSG00000071076	7427.17751	0.76557607	0.038076102	4.873820074	1.09E-06	8.16E-05

Supplementary table 5: Related to figure. 2f (Significant genes in LPS 30min compared to PBS)

	symbol	Gene	baseMean	log2FoldChange	lfcSE	stat	pvalue	padj
1	Icosl	ENSMUSG00000000732	1879.946841	1.504458575	0.063748802	14.50158352	1.18E-47	1.87E-45
2	Ccl3	ENSMUSG00000000982	404.881459	2.573510088	0.338845122	5.883248588	4.02E-09	2.82E-07
3	Hspb1	ENSMUSG00000004951	18265.25274	1.877508638	0.027849643	46.58977561	0	0
4	Mcoln2	ENSMUSG00000011008	367.7904762	-1.631015211	0.281752415	-3.730279339	0.000191268	0.008475666
5	Slamf6	ENSMUSG00000015314	8.200019969	-3.398079137	0.877148778	-3.21277212	0.001314605	0.049889051
6	Il10	ENSMUSG00000016529	26.51114318	6.728191752	1.265001615	4.86022443	1.17E-06	6.84E-05
7	Irf1	ENSMUSG00000018899	8354.069478	2.754300098	0.038793774	56.04765649	0	0
8	Ccl4	ENSMUSG00000018930	506.4697284	3.636719139	0.417237964	7.326081042	2.37E-13	2.08E-11
9	Tnfaip3	ENSMUSG00000019850	9466.877995	5.112839587	0.050863898	89.11703077	0	0
10	Rel	ENSMUSG00000020275	1083.526703	2.66826095	0.088148803	23.69017935	4.55E-124	1.16E-121
11	Nfkbia	ENSMUSG00000021025	24691.37012	2.597515239	0.0314116	64.22835	0	0
12	Rgcc	ENSMUSG00000022018	2726.351204	1.582905511	0.051410787	19.50768639	9.45E-85	1.85E-82
13	Acod1	ENSMUSG00000022126	471.3328772	3.019008041	0.697231333	3.498133153	0.000468527	0.019688014
14	Litaf	ENSMUSG00000022500	3643.467938	1.317907591	0.115779666	6.373378129	1.85E-10	1.41E-08
15	Noct	ENSMUSG00000023087	19075.1269	1.786656712	0.049364788	24.44367268	5.88E-132	1.59E-129
16	Tnf	ENSMUSG00000024401	274.3510962	7.476948949	0.472681179	14.59112241	3.20E-48	5.12E-46
17	Rela	ENSMUSG00000024927	4893.879484	0.922334974	0.047371564	7.226592197	4.95E-13	4.31E-11
18	Il6	ENSMUSG00000025746	3036.369636	6.195452576	1.204530641	4.661942492	3.13E-06	0.000177085
19	Hspd1	ENSMUSG00000025980	2345.995886	0.806270299	0.056689158	3.99142102	6.57E-05	0.003145544
20	Il1r1	ENSMUSG00000026072	7328.016784	0.955088447	0.033950889	11.04797136	2.24E-28	2.75E-26
21	Il1rn	ENSMUSG00000026981	784.2277529	4.381194878	1.052469318	3.611691869	0.000304206	0.013032727
22	Il1b	ENSMUSG00000027398	830.013287	5.071309407	0.228768058	19.63258967	8.15E-86	1.62E-83
23	Il1a	ENSMUSG00000027399	181.8071872	3.594829976	0.420630291	7.167410525	7.64E-13	6.60E-11
24	Tlr2	ENSMUSG00000027995	2660.837425	1.430201036	0.085689834	9.921842434	3.35E-23	3.78E-21
25	Bcl10	ENSMUSG00000028191	1122.860408	1.097619431	0.07728364	6.697658539	2.12E-11	1.71E-09
26	Nr4a3	ENSMUSG00000028341	505.4711915	2.774222829	0.146988934	14.92780962	2.17E-50	3.61E-48
27	Errfi1	ENSMUSG00000028967	11326.66887	1.690463081	0.050563312	21.96183434	6.67E-107	1.49E-104
28	Cxcl1	ENSMUSG00000029380	28342.61911	9.772424871	0.137969383	66.62655643	0	0

29	Clec4e	ENSMUSG00000030142	165.0834934	4.894203734	0.677775486	6.365240147	1.95E-10	1.48E-08
30	Ptgs2	ENSMUSG00000032487	32813.74795	4.710273664	0.040629405	101.6572518	0	0
31	Trib1	ENSMUSG00000032501	1427.060222	1.343238029	0.078522069	9.72004476	2.48E-22	2.77E-20
32	Nlrp3	ENSMUSG00000032691	233.0219436	2.433594939	0.223194147	8.304854597	9.99E-17	9.62E-15
33	P2ry2	ENSMUSG00000032860	632.2885949	1.058828499	0.106080735	4.513812029	6.37E-06	0.000346566
34	Cxcl10	ENSMUSG00000034855	51363.8536	4.218751018	0.122252666	29.76418539	1.14E-194	3.67E-192
35	Amh	ENSMUSG00000035262	45.20270334	1.864273919	0.354205528	3.625787344	0.000288082	0.012366119
36	Ccl12	ENSMUSG00000035352	349.1488707	4.646141295	0.244654521	16.61993117	5.00E-62	8.84E-60
37	Ccl2	ENSMUSG00000035385	3958.966572	4.742247163	0.236644089	17.58863777	3.01E-69	5.55E-67
38	Sbno2	ENSMUSG00000035673	8813.092372	1.123067615	0.040548965	13.39288485	6.65E-41	9.48E-39
39	Tnfsf9	ENSMUSG00000035678	109.0126621	3.228234027	0.374449957	7.072330972	1.52E-12	1.30E-10
40	Icam1	ENSMUSG00000037405	29157.6081	4.798017735	0.033141625	127.2725067	0	0
41	Serpine1	ENSMUSG00000037411	1108.96844	3.22429004	0.100707826	26.25704612	5.94E-152	1.76E-149
42	Arid5a	ENSMUSG00000037447	4936.326917	2.758623668	0.069013637	31.56801704	1.01E-218	3.59E-216
43	Gch1	ENSMUSG00000037580	2429.995259	1.416473305	0.063911581	13.08797702	3.86E-39	5.39E-37
44	Ddx60	ENSMUSG00000037921	1308.441645	-0.964823562	0.112576573	-3.418327194	0.000630073	0.025736786
45	Egr1	ENSMUSG00000038418	11656.38061	5.127535347	0.039328259	115.6302227	0	0
46	Akap12	ENSMUSG00000038587	43080.10233	1.224603757	0.028168542	22.88381652	6.73E-116	1.61E-113
47	Thbs1	ENSMUSG00000040152	3109.321826	0.947928939	0.057249752	6.426734236	1.30E-10	1.00E-08
48	Ripk2	ENSMUSG00000041135	1319.485889	1.172687208	0.09950038	5.956632589	2.57E-09	1.84E-07
49	Prkd2	ENSMUSG00000041187	2276.395672	0.77989865	0.058322486	3.427471358	0.000609231	0.024931852
50	Atf4	ENSMUSG00000042406	5738.951968	0.898136531	0.03783573	8.408362373	4.16E-17	4.05E-15
51	Zc3h12a	ENSMUSG00000042677	1163.744481	2.439703863	0.105298008	17.66133946	8.32E-70	1.56E-67
52	Zfp36	ENSMUSG00000044786	18255.99707	5.36220671	0.036246384	131.9361051	0	0
53	Trim16	ENSMUSG00000047821	1325.781937	0.85961342	0.064079163	4.363562286	1.28E-05	0.000668294
54	C5ar1	ENSMUSG00000049130	233.3220744	1.607206308	0.173967068	5.904602064	3.53E-09	2.49E-07
55	Trex1	ENSMUSG00000049734	1410.032733	1.050249214	0.086420365	5.441416647	5.29E-08	3.50E-06
56	Cd14	ENSMUSG00000051439	7579.922493	4.130935146	0.095928659	37.01641584	6.23E-300	2.49E-297
57	Cx3cr1	ENSMUSG00000052336	149.401536	-1.236052231	0.170012864	-3.85883876	0.000113927	0.005272131
58	Jun	ENSMUSG00000052684	10484.16994	3.415445587	0.037984906	74.64663962	0	0

59	Junb	ENSMUSG00000052837	15095.43776	4.779297401	0.040769667	103.0005316	0	0
60	Bcl3	ENSMUSG00000053175	1716.137156	1.882687321	0.115566107	11.27222637	1.80E-29	2.24E-27
61	Nod2	ENSMUSG00000055994	1137.687625	1.744765935	0.085448101	13.63126769	2.61E-42	3.84E-40
62	Cebpg	ENSMUSG00000056216	2934.115846	0.798737439	0.058515273	3.738125601	0.000185397	0.008232167
63	Cebpb	ENSMUSG00000056501	5094.091349	3.016514087	0.055001565	44.29899535	0	0
64	Cxcl2	ENSMUSG00000058427	8389.878604	9.280584959	0.283354639	30.7056379	4.79E-207	1.64E-204
65	Osm	ENSMUSG00000058755	23.80602464	2.740166002	0.661382245	3.266138484	0.001090249	0.042401801
66	Jund	ENSMUSG00000071076	7427.17751	1.248338655	0.037894183	17.63697232	1.28E-69	2.38E-67
67	Gbp5	ENSMUSG00000105504	24611.98318	1.105360729	0.055289156	9.502057296	2.06E-21	2.27E-19

Supplementary table 6: Related to figure. 2h

	symbol	gene	baseMean	log2FoldChange	lfcSE	stat	pvalue	padj
1	Klf4	ENSMUSG00000003032	5628.479158	1.561054452	0.039249817	24.99513455	6.91E-138	5.73E-135
2	Rgcc	ENSMUSG00000022018	2726.351204	1.391072291	0.051384252	15.78445258	3.98E-56	1.76E-53
3	Adamts1	ENSMUSG00000022893	16433.79349	1.898210089	0.032084745	41.08525936	0	0
4	Xdh	ENSMUSG00000024066	10150.73315	0.801509034	0.044141597	5.018147302	5.22E-07	4.14E-05
5	Creb3l1	ENSMUSG00000027230	311.2062805	-1.053438381	0.14073789	-3.36397243	0.000768292	0.036555603
6	Angpt4	ENSMUSG00000027460	19.28326026	-2.804211367	0.528323619	-4.209941194	2.55E-05	0.001619911
7	Sema4a	ENSMUSG00000028064	548.2476075	1.038197397	0.105836351	4.329300767	1.50E-05	0.000975637
8	Adgrb2	ENSMUSG00000028782	2309.912385	1.187971288	0.086566445	7.023174946	2.17E-12	2.81E-10
9	Adgrb1	ENSMUSG00000034730	5634.586257	1.034612651	0.049206819	9.238814081	2.49E-20	5.22E-18
10	Cxcl10	ENSMUSG00000034855	51363.8536	1.080808763	0.142671141	3.510231704	0.000447716	0.022690504
11	Tgfb2	ENSMUSG00000039239	1720.669867	-1.078693121	0.063313852	-7.876524781	3.37E-15	5.21E-13
12	Abcc8	ENSMUSG00000040136	298.3627786	1.441290513	0.214336351	4.018406154	5.86E-05	0.003511016
13	Klf2	ENSMUSG00000055148	17486.56426	1.787370514	0.025624093	47.11856527	0	0

Supplementary table 7: Related to figure. 2i (Significant genes in LPS 30min compared to PBS)

	symbol	gene	baseMean	log2FoldChange	lfcSE	stat	pvalue	padj
1	Alox12	ENSMUSG00000000320	317.3473577	-1.238545462	0.126138344	-5.22081898	1.78E-07	1.13E-05
2	Tnf	ENSMUSG00000024401	274.3510962	7.476948949	0.472681179	14.59112241	3.20E-48	5.12E-46
3	Ptgs2	ENSMUSG00000032487	32813.74795	4.710273664	0.040629405	101.6572518	0	0
4	Manf	ENSMUSG00000032575	2123.97003	0.80372198	0.058282624	3.838570858	0.000123753	0.005678894
5	P2ry2	ENSMUSG00000032860	632.2885949	1.058828499	0.106080735	4.513812029	6.37E-06	0.000346566
6	Dusp5	ENSMUSG00000034765	313.5667066	3.641002673	0.153367445	19.95862072	1.26E-88	2.59E-86
7	Icam1	ENSMUSG00000037405	29157.6081	4.798017735	0.033141625	127.2725067	0	0
8	Gch1	ENSMUSG00000037580	2429.995259	1.416473305	0.063911581	13.08797702	3.86E-39	5.39E-37
9	Kcna5	ENSMUSG00000045534	1146.58785	1.817064797	0.086049858	14.37613987	7.31E-47	1.14E-44
10	Per2	ENSMUSG00000055866	1530.504748	2.58010422	0.074760952	26.75332718	1.13E-157	3.39E-155
11	Kdr	ENSMUSG00000062960	4464.77845	-1.019209275	0.036781665	-11.94098391	7.24E-33	9.23E-31
12	Gpx1	ENSMUSG00000063856	7074.013933	1.513798805	0.0362497	25.76018028	2.48E-146	7.06E-144

Supplementary table 8: Related to supplementary figure. 2f (Significant genes in LPS 4hrs compared to PBS)

	symbol	Gene	baseMean	log2FoldChange	lfcSE	stat	pvalue	padj
1	Rock2	ENSMUSG00000020580	3758.17721	1.036686543	0.049498154	9.226334822	2.80E-20	3.67E-19
2	Akap11	ENSMUSG00000022016	2627.730733	1.613012618	0.060526514	17.06710911	2.61E-65	6.31E-64
3	Cldn1	ENSMUSG00000022512	864.6534226	1.780373756	0.074041812	16.21210659	4.14E-59	9.42E-58
4	Tnf	ENSMUSG00000024401	274.3510962	7.705633286	0.47324616	15.05692784	3.11E-51	6.58E-50
5	Il1b	ENSMUSG00000027398	830.013287	7.772446081	0.226232124	31.79232891	8.26E-222	4.32E-220
6	Abcb1b	ENSMUSG00000028970	328.3272325	2.583478401	0.266590107	7.515201601	5.68E-14	6.17E-13
7	Add1	ENSMUSG00000029106	3748.931222	-1.140493974	0.07114637	-7.87804038	3.33E-15	3.77E-14
8	Tnfrsf1a	ENSMUSG00000030341	3178.189208	1.752788543	0.041417602	28.31618665	2.18E-176	9.59E-175
9	Msn	ENSMUSG00000031207	13388.79528	1.621256529	0.027567151	37.77164121	0	0
10	Cdh5	ENSMUSG00000031871	17510.59564	1.939112582	0.023559715	57.68798907	0	0
11	Myd88	ENSMUSG00000032508	2131.672384	3.448350873	0.052121016	55.0325201	0	0
12	Icam1	ENSMUSG00000037405	29157.6081	5.737082798	0.033101335	155.7968234	0	0
13	Ppp1r16b	ENSMUSG00000037754	1373.950257	-2.046347194	0.084805321	-17.29074508	5.52E-67	1.35E-65
14	F11r	ENSMUSG00000038235	4517.130126	1.658245782	0.0501587	21.49668523	1.67E-102	5.30E-101
15	Cldn5	ENSMUSG00000041378	30428.70436	-2.33273169	0.024128197	-72.6424649	0	0
16	Sox18	ENSMUSG00000046470	5602.589436	-4.392841692	0.062353899	-61.1484084	0	0
17	Rap2c	ENSMUSG00000050029	696.1819687	1.09626283	0.089976966	5.737722147	9.60E-09	8.35E-08
18	Ezr	ENSMUSG00000052397	4317.461626	1.261849832	0.041061805	16.60545206	6.36E-62	1.49E-60
19	Rap1b	ENSMUSG00000052681	5501.669084	1.614504233	0.036870195	28.05800834	3.19E-173	1.39E-171
20	Rapgef2	ENSMUSG00000062232	3063.862558	0.969347635	0.078068148	4.987279028	6.12E-07	4.74E-06
21	S1pr3	ENSMUSG00000067586	1641.50632	1.027696018	0.061989425	7.222135334	5.12E-13	5.38E-12
22	Afdn	ENSMUSG00000068036	3429.291756	-0.916680434	0.056710912	-5.936783953	2.91E-09	2.62E-08
23	Pde2a	ENSMUSG00000110195	6427.670728	-1.75261646	0.090581079	-12.94549011	2.49E-38	4.49E-37

Supplementary table 9a: Related to figure. 4i (Significant genes in CECs LPS 30min compared to CECs PBS)

	symbol	Gene	baseMean	log2FoldChange	lfcSE	stat	pvalue	padj
1	Ier3	ENSMUSG00000003541	8135.428722	2.336973656	0.292535323	7.988688796	1.36E-15	1.91E-12
2	Fosb	ENSMUSG00000003545	219.4164628	2.309806057	0.587907419	3.928860193	8.53E-05	0.009096738
3	Fos	ENSMUSG00000021250	5687.064247	4.209372136	0.303381489	13.8748483	9.00E-44	3.03E-40
4	Nr4a1	ENSMUSG00000023034	5131.201155	1.930970851	0.570182562	3.386583488	0.000707687	0.039331537
5	Dusp1	ENSMUSG00000024190	16564.87115	2.154312343	0.36404657	5.917683398	3.27E-09	2.04E-06
6	Atf3	ENSMUSG00000026628	4560.245227	1.799667051	0.369939016	4.864766827	1.15E-06	0.000321621
7	Egr3	ENSMUSG00000033730	570.0468307	2.526006567	0.729129483	3.464414243	0.000531388	0.0329063
8	Egr1	ENSMUSG00000038418	12809.86023	5.238246897	0.386420953	13.55580451	7.32E-42	2.05E-38
9	Jun	ENSMUSG00000052684	11671.66241	1.265590318	0.258361775	4.898519978	9.66E-07	0.000285279
10	Junb	ENSMUSG00000052837	13748.22188	3.973721717	0.188333679	21.09936867	8.06E-99	1.36E-94
11	Ier2	ENSMUSG00000053560	8088.842212	2.331138913	0.249676643	9.336631906	9.94E-21	2.09E-17
12	Hspa1a	ENSMUSG00000091971	2661.949302	3.794901738	0.762873947	4.974480718	6.54E-07	0.000216023

Supplementary table 9b: Related to figure. 4i (Significant genes in CECs LPS 1hr compared to CECs PBS)

	symbol	gene	baseMean	log2FoldChange	lfcSE	stat	pvalue	padj
1	Ier3	ENSMUSG00000003541	8135.428722	4.316206651	0.292386187	14.76200603	2.58E-49	3.10E-46
2	Fosb	ENSMUSG00000003545	219.4164628	4.365501174	0.585844574	7.451637122	9.22E-14	2.25E-11
3	Fos	ENSMUSG00000021250	5687.064247	4.69781694	0.303330732	15.48744142	4.22E-54	5.46E-51
4	Nr4a1	ENSMUSG00000023034	5131.201155	3.280229	0.570143371	5.753340597	8.75E-09	1.23E-06
5	Dusp1	ENSMUSG00000024190	16564.87115	2.388631018	0.364042368	6.561409416	5.33E-11	9.97E-09
6	Atf3	ENSMUSG00000026628	4560.245227	3.48503994	0.369720016	9.426159763	4.25E-21	1.71E-18
7	Egr3	ENSMUSG00000033730	570.0468307	4.826944369	0.728558975	6.625331012	3.46E-11	6.63E-09
8	Egr1	ENSMUSG00000038418	12809.86023	5.998727078	0.386402528	15.52455441	2.37E-54	3.32E-51
9	Jun	ENSMUSG00000052684	11671.66241	0.898878533	0.258386861	3.478808985	0.000503647	0.017309026
10	Junb	ENSMUSG00000052837	13748.22188	5.051029738	0.188256341	26.83059556	1.42E-158	2.39E-154
11	Ier2	ENSMUSG00000053560	8088.842212	3.059192794	0.249633889	12.25471752	1.59E-34	1.33E-31
12	Hspa1a	ENSMUSG00000091971	2661.949302	2.911758218	0.762907353	3.816660315	0.00013527	0.006355915

Supplementary table 9c: Related to figure. 4i (Significant genes in Microglia LPS 1hr compared to Microglia PBS)

	symbol	Gene	baseMean	log2FoldChange	lfcSE	stat	pvalue	padj
1	Ier3	ENSMUSG00000003541	8135.428722	1.167898781	0.303572978	3.847176353	0.000119487	0.040820259
2	Dusp1	ENSMUSG00000024190	16564.87115	1.816826788	0.364844929	4.979723285	6.37E-07	0.000493788

Supplementary table 9d: Related to figure. 4i (Significant genes in CECs LPS 2hrs compared to CECs PBS)

	symbol	Gene	baseMean	log2FoldChange	lfcSE	stat	pvalue	padj
1	Ier3	ENSMUSG00000003541	8135.428722	4.683655051	0.292398145	16.01807373	9.56E-58	2.00E-55
2	Fosb	ENSMUSG00000003545	219.4164628	3.48459486	0.587061097	5.93565964	2.93E-09	5.29E-08
3	Fos	ENSMUSG00000021250	5687.064247	5.738916731	0.303307107	18.92114163	7.64E-80	3.15E-77
4	Nr4a1	ENSMUSG00000023034	5131.201155	1.419692743	0.570257961	2.489562337	0.012790049	0.047983168
5	Atf3	ENSMUSG00000026628	4560.245227	4.823369651	0.369686364	13.04719385	6.59E-39	7.01E-37
6	Egr3	ENSMUSG00000033730	570.0468307	2.727501882	0.729372093	3.739520483	0.000184372	0.001317063
7	Egr1	ENSMUSG00000038418	12809.86023	5.371384782	0.386438245	13.89972358	6.36E-44	8.29E-42
8	Jun	ENSMUSG00000052684	11671.66241	0.964812394	0.258458147	3.732954076	0.000189247	0.001348288
9	Junb	ENSMUSG00000052837	13748.22188	5.070908527	0.188294284	26.93076192	9.59E-160	2.08E-156
10	Ier2	ENSMUSG00000053560	8088.842212	2.717994436	0.249694978	10.88525874	1.36E-27	9.33E-26

Supplementary table 9e: Related to figure. 4i (Significant genes in Microglia LPS 2hrs compared to Microglia PBS)

	symbol	Gene	baseMean	log2FoldChange	lfcSE	stat	pvalue	padj
1	Ier3	ENSMUSG00000003541	8135.428722	5.861828001	0.300134497	19.53067064	6.02E-85	4.62E-81
2	Fos	ENSMUSG00000021250	5687.064247	2.475565632	0.302216671	8.191360284	2.58E-16	4.50E-14
3	Nr4a1	ENSMUSG00000023034	5131.201155	3.128970741	0.577905919	5.41432548	6.15E-08	4.18E-06
4	Dusp1	ENSMUSG00000024190	16564.87115	4.207821414	0.364691633	11.53802564	8.49E-31	3.17E-28
5	Egr3	ENSMUSG00000033730	570.0468307	2.384895347	0.770700634	3.094450997	0.001971775	0.046815922
6	Egr1	ENSMUSG00000038418	12809.86023	4.119458749	0.385892396	10.67514882	1.33E-26	4.00E-24
7	Junb	ENSMUSG00000052837	13748.22188	2.84917422	0.187051031	15.23206903	2.17E-52	2.56E-49
8	Ier2	ENSMUSG00000053560	8088.842212	4.315333951	0.251993964	17.12475128	9.70E-66	2.13E-62
9	Hspa1a	ENSMUSG00000091971	2661.949302	-2.675590888	0.765105934	-3.497020177	0.000470486	0.014066897

Supplementary table 10: Related to figure. 4j

	symbol	gene	baseMean	log2FoldChange	lfcSE	stat	pvalue	padj
1	Tnfaip3	ENSMUSG00000019850	7910.386742	2.381939944	0.40323561	5.907067442	3.48E-09	2.09E-06
2	Nfkbia	ENSMUSG00000021025	27835.28986	1.186108274	0.23952242	4.951971823	7.35E-07	0.000237807
3	Litaf	ENSMUSG00000022500	7770.491065	1.5357449	0.206962037	7.420418375	1.17E-13	1.31E-10
4	Tnf	ENSMUSG00000024401	1042.572106	5.377209529	0.692324133	7.76689599	8.04E-15	1.04E-11
5	Cxcl1	ENSMUSG00000029380	8769.124715	5.911435088	0.366120477	16.14614711	1.21E-58	6.79E-55
6	Arid5a	ENSMUSG00000037447	6760.818824	0.781413651	0.221836169	3.522480819	0.000427528	0.028569727
7	Gch1	ENSMUSG00000037580	3923.860293	0.943525179	0.245093601	3.849652431	0.000118286	0.012072295
8	Zfp36	ENSMUSG00000044786	17516.75061	3.890691681	0.190475043	20.42625441	9.77E-93	8.23E-89
9	Jun	ENSMUSG00000052684	11671.66241	1.265590318	0.258361775	4.898519978	9.66E-07	0.000285279
10	Junb	ENSMUSG00000052837	13748.22188	3.973721717	0.188333679	21.09936867	8.06E-99	1.36E-94
11	Cxcl2	ENSMUSG00000058427	926.0697058	5.359533649	0.579689326	9.245527583	2.34E-20	4.38E-17
12	Irak2	ENSMUSG00000060477	4961.489113	1.055770215	0.1643567	6.423651819	1.33E-10	1.12E-07

Supplementary table 11: Related to figure. 4k

	symbol	gene	baseMean	log2FoldChange	lfcSE	stat	pvalue	padj
1	Tgfb1	ENSMUSG00000002603	2507.480189	1.059087022	0.13798266	7.675508057	1.65E-14	4.34E-12
2	Relb	ENSMUSG00000002983	1340.526405	0.854292574	0.21260686	4.018179717	5.86E-05	0.003217123
3	Bcr	ENSMUSG00000009681	1844.242677	1.60849972	0.416896267	3.858273264	0.000114191	0.005639224
4	Hyal2	ENSMUSG00000010047	1985.612833	0.586900663	0.131349376	4.468240951	7.89E-06	0.000606436
5	Irf1	ENSMUSG00000018899	15271.61072	2.809774987	0.15375579	18.27427114	1.33E-74	3.72E-71
6	Tnfaip3	ENSMUSG00000019850	7910.386742	4.824742281	0.402928175	11.97419933	4.85E-33	3.71E-30
7	Rel	ENSMUSG00000020275	1237.461989	2.927779886	0.337268209	8.680865291	3.93E-18	1.30E-15
8	Nfkbia	ENSMUSG00000021025	27835.28986	2.13622235	0.239496186	8.919650804	4.68E-19	1.68E-16
9	Edn1	ENSMUSG00000021367	2259.61847	1.493533582	0.3083393	4.843798966	1.27E-06	0.000112899
10	Clu	ENSMUSG00000022037	3821.713187	-0.980824386	0.316666189	-3.097344841	0.001952626	0.046575381
11	Litaf	ENSMUSG00000022500	7770.491065	2.208903877	0.20669228	10.68692005	1.17E-26	6.37E-24
12	Noct	ENSMUSG00000023087	4679.07607	2.792337948	0.240463924	11.61229469	3.57E-31	2.50E-28
13	Tnf	ENSMUSG00000024401	1042.572106	7.856960417	0.686295555	11.44836267	2.40E-30	1.49E-27
14	Rela	ENSMUSG00000024927	6819.144291	0.99936955	0.137605151	7.262588232	3.80E-13	8.64E-11
15	Nfkb2	ENSMUSG00000025225	1853.369785	1.153515503	0.253946034	4.542364704	5.56E-06	0.000437735
16	Alpk1	ENSMUSG00000028028	128.4493761	2.444182093	0.742308775	3.292675738	0.000992389	0.028373215
17	Bcl10	ENSMUSG00000028191	1749.916425	1.400044019	0.103262245	13.55814043	7.09E-42	7.96E-39
18	Nr4a3	ENSMUSG00000028341	96.09023724	3.794400712	0.85919269	4.41623952	1.00E-05	0.000758425
19	Errfi1	ENSMUSG00000028967	2397.684275	2.524757026	0.305552109	8.262934386	1.42E-16	4.27E-14
20	Prkcz	ENSMUSG00000029053	70.47244279	-1.708238858	0.530200938	-3.221870683	0.001273566	0.034260147
21	Cxcl1	ENSMUSG00000029380	8769.124715	9.391318365	0.365634199	25.68501084	1.72E-145	9.65E-142
22	Cx3cl1	ENSMUSG00000031778	7029.169566	1.334806587	0.392256534	3.402891911	0.000666767	0.021428146
23	Birc3	ENSMUSG00000032000	2369.327228	1.563963041	0.280805923	5.569551464	2.55E-08	3.33E-06
24	Ptgs2	ENSMUSG00000032487	4963.684051	3.265914323	0.619988566	5.267700893	1.38E-07	1.61E-05
25	P2ry2	ENSMUSG00000032860	1119.019686	1.303398894	0.352183459	3.700908885	0.000214829	0.009156322
26	Cxcl10	ENSMUSG00000034855	27643.5159	3.678949719	0.505030987	7.284601965	3.23E-13	7.44E-11
27	Sbno2	ENSMUSG00000035673	6547.703709	1.650526882	0.211230389	7.813870378	5.55E-15	1.51E-12
28	Nfkbid	ENSMUSG00000036931	581.4950379	7.537214177	0.482380838	15.62502817	4.92E-55	7.53E-52

29	Icam1	ENSMUSG00000037405	43731.10471	4.686395468	0.234291155	20.00244297	5.24E-89	1.77E-85
30	Serpine1	ENSMUSG00000037411	66.32557586	4.823362092	0.950030401	5.077060784	3.83E-07	3.98E-05
31	Arid5a	ENSMUSG00000037447	6760.818824	1.887416993	0.221554406	8.518977465	1.61E-17	5.11E-15
32	Gch1	ENSMUSG00000037580	3923.860293	1.048962695	0.245067139	4.280307431	1.87E-05	0.00128809
33	Egr1	ENSMUSG00000038418	12809.86023	5.998727078	0.386402528	15.52455441	2.37E-54	3.32E-51
34	Akap12	ENSMUSG00000038587	18875.22354	1.141460079	0.360619148	3.165278622	0.001549345	0.039955546
35	Ripk2	ENSMUSG00000041135	1062.526998	1.212675819	0.276350286	4.388183701	1.14E-05	0.000836884
36	Atf4	ENSMUSG00000042406	1958.857838	1.026950851	0.14688433	6.991561683	2.72E-12	5.58E-10
37	Zc3h12a	ENSMUSG00000042677	489.2115939	3.799972203	0.299478976	12.6886109	6.84E-37	6.40E-34
38	Zfp36	ENSMUSG00000044786	17516.75061	5.004977939	0.190426562	26.28298221	3.00E-152	2.53E-148
39	Tifa	ENSMUSG00000046688	2287.128138	2.084513907	0.256859329	8.11539108	4.84E-16	1.38E-13
40	Trex1	ENSMUSG00000049734	2840.854999	2.314923096	0.192800518	12.00683029	3.27E-33	2.62E-30
41	Jun	ENSMUSG00000052684	11671.66241	0.898878533	0.258386861	3.478808985	0.000503647	0.017309026
42	Junb	ENSMUSG00000052837	13748.22188	5.051029738	0.188256341	26.83059556	1.42E-158	2.39E-154
43	Bcl3	ENSMUSG00000053175	1950.996273	1.98766244	0.277745028	7.15642851	8.28E-13	1.81E-10
44	Nod2	ENSMUSG00000055994	152.6489868	2.602853473	0.535978652	4.856263326	1.20E-06	0.000107723
45	Cxcl2	ENSMUSG00000058427	926.0697058	9.34911204	0.575419051	16.24748438	2.33E-59	4.35E-56
46	Cxcl11	ENSMUSG00000060183	1006.579109	2.717911833	0.583923996	4.654564382	3.25E-06	0.000269329
47	Irak2	ENSMUSG00000060477	4961.489113	1.396951018	0.164213675	8.506910402	1.79E-17	5.57E-15
48	Jund	ENSMUSG00000071076	3952.106918	1.083051938	0.206580068	5.242770754	1.58E-07	1.79E-05

Supplementary table 12: Related to supplementary figure. 4d (Significant genes in Microglia LPS 1hr compared to Microglia PBS)

	symbol	gene	baseMean	log2FoldChange	lfcSE	stat	pvalue	padj
1	Nfkbia	ENSMUSG00000021025	27835.28986	1.572368413	0.240371502	6.541409457	6.09E-11	1.55E-07
2	Tnf	ENSMUSG00000024401	1042.572106	2.718076665	0.439467669	6.184929766	6.21E-10	1.39E-06
3	Il1b	ENSMUSG00000027398	2717.403984	2.848321268	0.571237158	4.986232479	6.16E-07	0.000493788
4	Cxcl1	ENSMUSG00000029380	8769.124715	7.405799174	1.07762474	6.872335885	6.32E-12	1.88E-08
5	Ptgs2	ENSMUSG00000032487	4963.684051	4.382767045	0.862765525	5.079905162	3.78E-07	0.000320728
6	Cxcl10	ENSMUSG00000034855	27643.5159	2.247146779	0.503956425	4.459010077	8.23E-06	0.005439257
7	Cxcl2	ENSMUSG00000058427	926.0697058	4.895931982	0.697472926	7.01952979	2.23E-12	9.22E-09

Supplementary table 13: Related to figure. 4o

	symbol	gene	baseMean	log2FoldChange	lfcSE	stat	pvalue	padj
1	Hpn	ENSMUSG00000001249	89.13264462	-4.926243148	1.298193429	-3.794691175	0.000147827	0.013988991
2	Pmp22	ENSMUSG00000018217	7785.551826	0.614641195	0.134032113	4.585775625	4.52E-06	0.001062378
3	Irf1	ENSMUSG00000018899	15271.61072	0.829160099	0.154150522	5.378899063	7.49E-08	3.16E-05
4	Tnfaip3	ENSMUSG00000019850	7910.386742	2.381939944	0.40323561	5.907067442	3.48E-09	2.09E-06
5	Nf1	ENSMUSG00000020716	236.7811338	1.681174979	0.446483956	3.765364818	0.000166306	0.015199055
6	Dusp1	ENSMUSG00000024190	16564.87115	2.154312343	0.36404657	5.917683398	3.27E-09	2.04E-06
7	Tnf	ENSMUSG00000024401	1042.572106	5.377209529	0.692324133	7.76689599	8.04E-15	1.04E-11
8	Ptprc	ENSMUSG00000026395	3057.618869	1.890922999	0.525068059	3.601291235	0.000316641	0.024117919
9	Mar7	ENSMUSG00000026977	1061.994783	-0.666796392	0.168482753	-3.957653719	7.57E-05	0.008385608
10	Pde3b	ENSMUSG00000030671	5281.451887	2.183187118	0.580621882	3.760084123	0.000169856	0.015296144
11	Plxnb3	ENSMUSG00000031385	196.9264164	-4.758352495	1.245197044	-3.821365076	0.000132715	0.013303101
12	Dusp5	ENSMUSG00000034765	293.8495755	2.991254662	0.457487969	6.5384335	6.22E-11	5.51E-08
13	C1qa	ENSMUSG00000036887	35905.89582	1.254550087	0.229437985	5.467926723	4.55E-08	2.07E-05
14	C1qc	ENSMUSG00000036896	51400.31892	1.152980796	0.311178398	3.705208338	0.000211217	0.017826638
15	C1qb	ENSMUSG00000036905	64973.93876	0.923717915	0.276973229	3.335044036	0.000852859	0.044881701
16	Nfkbid	ENSMUSG00000036931	581.4950379	4.794383885	0.485665253	9.871786905	5.52E-23	1.33E-19
17	Icam1	ENSMUSG00000037405	43731.10471	0.926677201	0.234798733	3.946687403	7.92E-05	0.008664935
18	Gch1	ENSMUSG00000037580	3923.860293	0.943525179	0.245093601	3.849652431	0.000118286	0.012072295
19	Ctss	ENSMUSG00000038642	59580.50645	1.284049935	0.295781457	4.34121174	1.42E-05	0.002386213
20	Rnd1	ENSMUSG00000054855	4907.485517	1.607720828	0.391120644	4.110549651	3.95E-05	0.005152758

Supplementary table 14: Related to supplementary figure. 4y (Significant genes in CECs LPS 2hrs compared to CECs PBS)

	symbol	gene	baseMean	log2FoldChange	lfcSE	stat	pvalue	padj
1	Mapk7	ENSMUSG00000001034	580.6858303	-1.612498273	0.34426695	-4.683860217	2.82E-06	3.13E-05
2	Angptl4	ENSMUSG00000002289	765.7255422	1.950641689	0.463477179	4.208711406	2.57E-05	0.000230882
3	Hipk1	ENSMUSG00000008730	1225.084767	0.925725775	0.268436379	3.448585395	0.000563531	0.003513914
4	Gata2	ENSMUSG00000015053	2286.950443	-2.047916162	0.227095151	-9.017877086	1.92E-19	8.73E-18
5	Nfe2l2	ENSMUSG00000015839	4150.173826	1.43381944	0.114516219	12.520667	5.75E-36	5.70E-34
6	H2-M3	ENSMUSG00000016206	820.2550881	1.153458563	0.232326236	4.964822679	6.88E-07	8.60E-06
7	Cd40	ENSMUSG00000017652	984.1722645	3.096348537	0.427209051	7.247853308	4.23E-13	1.17E-11
8	Tnfaip3	ENSMUSG00000019850	7910.386742	5.714406435	0.402919648	14.18249636	1.18E-45	1.67E-43
9	Xbp1	ENSMUSG00000020484	6690.955555	1.739368133	0.121218698	14.34900856	1.08E-46	1.62E-44
10	Rock2	ENSMUSG00000020580	2519.091824	0.74546316	0.217128206	3.433285677	0.000596313	0.003689144
11	Marveld2	ENSMUSG00000021636	237.6618284	0.901914598	0.306460949	2.943000084	0.003250483	0.0155795
12	Rgcc	ENSMUSG00000022018	2797.369179	1.784736267	0.307889427	5.796679296	6.76E-09	1.16E-07
13	Rapgef3	ENSMUSG00000022469	2730.359467	1.553971258	0.31777902	4.890100232	1.01E-06	1.22E-05
14	Rock1	ENSMUSG00000024290	1699.135833	0.727506264	0.135639106	5.363543632	8.16E-08	1.20E-06
15	Tnf	ENSMUSG00000024401	1042.572106	7.749220036	0.687219918	11.2761866	1.72E-29	1.32E-27
16	Tjp2	ENSMUSG00000024812	2091.690126	0.783721692	0.205967594	3.805072819	0.000141762	0.001050557
17	Abl2	ENSMUSG00000026596	411.0228656	2.110738509	0.448870718	4.702330593	2.57E-06	2.89E-05
18	Il1b	ENSMUSG00000027398	2717.403984	4.676412775	0.619715314	7.546066185	4.49E-14	1.36E-12
19	Tnfrsf1a	ENSMUSG00000030341	3278.782048	1.129434837	0.134648874	8.388000576	4.94E-17	1.93E-15
20	Pak4	ENSMUSG00000030602	675.9973474	0.971402377	0.231837366	4.190016456	2.79E-05	0.000248817
21	Msn	ENSMUSG00000031207	15819.01783	0.779151526	0.221829448	3.512389954	0.000444096	0.002850136
22	Ikbkb	ENSMUSG00000031537	1880.504538	0.79292622	0.213689362	3.710649014	0.000206729	0.001457861
23	Myd88	ENSMUSG00000032508	2807.815602	4.488791382	0.207131708	21.67119379	3.84E-104	2.89E-101
24	Ccl12	ENSMUSG00000035352	2596.325135	5.590246843	0.548888767	10.1846625	2.32E-24	1.35E-22
25	Icam1	ENSMUSG00000037405	43731.10471	7.609493408	0.234260715	32.48301114	1.85E-231	1.07E-227
26	Serpine1	ENSMUSG00000037411	66.32557586	6.90205129	0.945622916	7.29894673	2.90E-13	8.15E-12
27	F11r	ENSMUSG00000038235	13419.00546	0.688231479	0.085773999	8.023777476	1.03E-15	3.58E-14
28	Rapgef1	ENSMUSG00000039844	1534.432818	1.293862028	0.203064389	6.37168357	1.87E-10	3.88E-09

29	Thbs1	ENSMUSG00000040152	744.4556348	3.642389916	0.502365407	7.250479165	4.15E-13	1.15E-11
30	Cldn5	ENSMUSG00000041378	25242.63569	-1.246527436	0.255827362	-4.872533681	1.10E-06	1.33E-05
31	Sox18	ENSMUSG00000046470	4160.119484	-2.183144597	0.300484856	-7.265406401	3.72E-13	1.03E-11
32	Ndnf	ENSMUSG00000049001	1523.065355	-2.597501481	0.510313148	-5.090014807	3.58E-07	4.74E-06
33	Rap1b	ENSMUSG00000052681	11441.10811	1.307163531	0.095296097	13.7168632	8.05E-43	1.00E-40
34	Gper1	ENSMUSG00000053647	548.0492641	-1.958672915	0.315904786	-6.200200191	5.64E-10	1.10E-08
35	Tnip2	ENSMUSG00000059866	721.1379561	1.007795425	0.194177756	5.190066281	2.10E-07	2.90E-06
36	Kdr	ENSMUSG00000062960	8316.589914	-1.353880163	0.316855488	-4.27286322	1.93E-05	0.000178788
37	Bmpr2	ENSMUSG00000067336	1631.045543	0.815998616	0.199240505	4.095545808	4.21E-05	0.000358371
38	Afdn	ENSMUSG00000068036	1258.247632	0.947656401	0.280885892	3.373812739	0.000741348	0.004451424
39	Myadm	ENSMUSG00000068566	5069.47159	0.695229586	0.227192237	3.060093934	0.002212676	0.011301918

References:

1. Boulay AC, Saubamea B, Decleves X, Cohen-Salmon M. Purification of Mouse Brain Vessels. *J Vis Exp* 2015; (105)**:** e53208.

2. Lee YK, Uchida H, Smith H, Ito A, Sanchez T. The isolation and molecular characterization of cerebral microvessels. *Nat Protoc* 2019; **14**(11)**:** 3059-3081.

3. Swartzlander DB, Propson NE, Roy ER, Saito T, Saido T, Wang B *et al.* Concurrent cell type–specific isolation and profiling of mouse brains in inflammation and Alzheimer's disease. *JCI Insight* 2018; **3**(13).
