# Supplementary material for: Temporal unsnarling of brain’s acute neuroinflammatory transcriptional profiles reveals panendothelitis as the earliest event preceding microgliosis
Source: Mol Psychiatry. 2020 Dec 8;26(8):3905–19. doi: 10.1038/s41380-020-00955-5 (PMC7722246; doi:10.1038/s41380-020-00955-5)
Supplement: Supplementary file 2 — Supplementary figures 1 through 5 [file 41380_2020_955_MOESM2_ESM.pdf]

Supplementary figure. 1

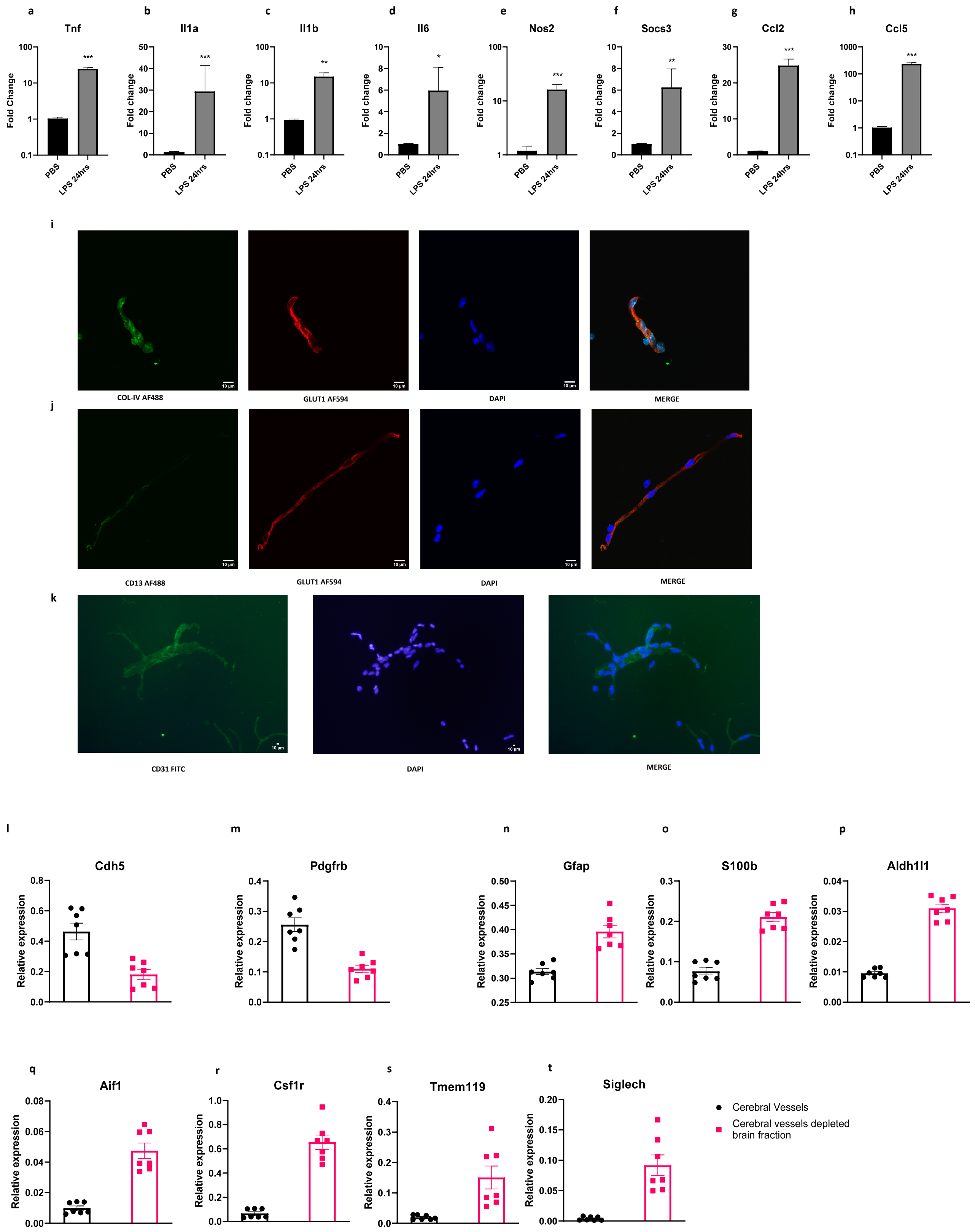

Supplementary figure. 1. Related to Figure.1. Cerebral vessels elicit proinflammatory responses during SAE.

a-h. mRNA fold change levels in whole forebrain lysates from the mice injected with 10mg/kg LPS for 24 hours, *Tnf, Il1a, Il1b, Il6, Nos2, Socs3, Ccl2* and *Ccl5* (n = 6-8 (male and female) mice in each group, Mann-Whitney test, *p<0.05, **p<0.01, ***p<0.001). i-k. Confocal images of immuno-stained isolated cerebral vessels for endothelial cell markers COL-IV, GLUT1, CD13 and CD31, (i) red channel is GlUT1, green channel is COL-IV, and blue channel is DAPI (j) red channel is GlUT1, green channel is CD13 and blue channel is DAPI, (k) green channel is CD31, blue channel is DAPI . (n=3 male mice). l-t. RT-qPCR analysis confirmed that the isolated cerebral vessels are enriched for expressing vasculature specific markers compared to the cerebral vessel depleted fractions, (l) *Cdh1*, (m) *Pdgfrb* (n-p) *Gfap, S100b* and *Aldh1l1* (q-t) *Aif1*, *Csf1r*, *Tmem119* and *Siglech*, (n= 6-7 males in each group, Bars represent mean ± SEM)

Supplementary figure. 2

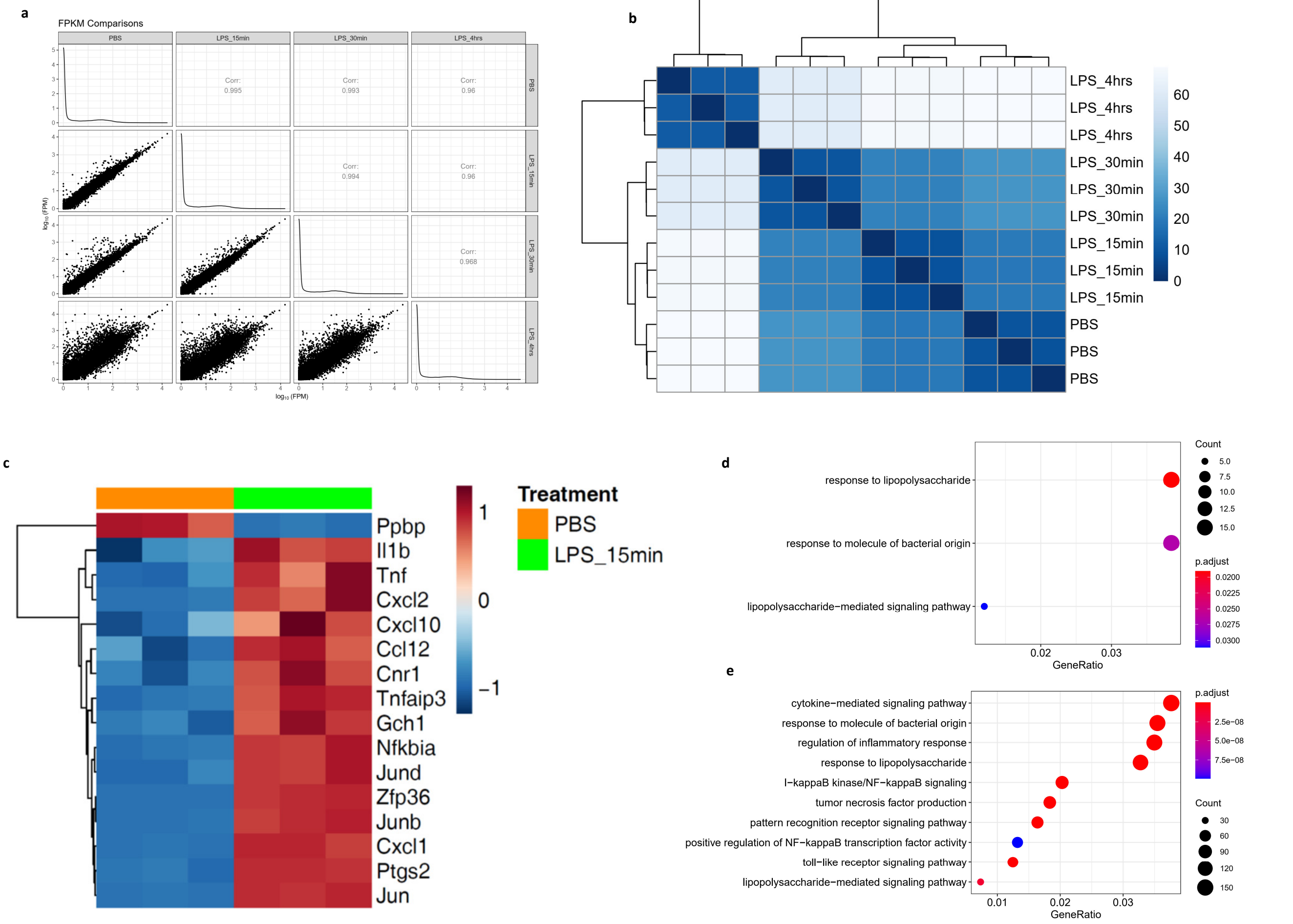

Supplementary figure. 2. Related to Figure. 2. Acute systemic inflammation rapidly affects the cerebral vessels during the initiation of SAE.

a. Scatterplot matrices showing the FPKM distributions (histograms) and correlation (Corr) values of the RNA-seq data from the cerebral vessels isolated from the brains of mice treated with 10mg/kg LPS for 15 minutes, 30 minutes, 4 hours and PBS. b. Unsupervised hierarchical clustering showing the sample-to-sample distances. c. Heatmap showing the significant (p.adj < 0.05 and LFC > 1.5) genes belonging to gene ontology (GO) term response to lipopolysaccharide (GO:0032496), in the cerebral vessels from the LPS treatment time points 15 minutes. d-e. GO enrichment dot plots showing the number of genes affected and the top 10 enriched inflammation related GO terms in the cerebral vessels from (d) 15 minutes and (e) 4 hours LPS treatment compared to PBS.

Supplementary figure. 2 continued

f

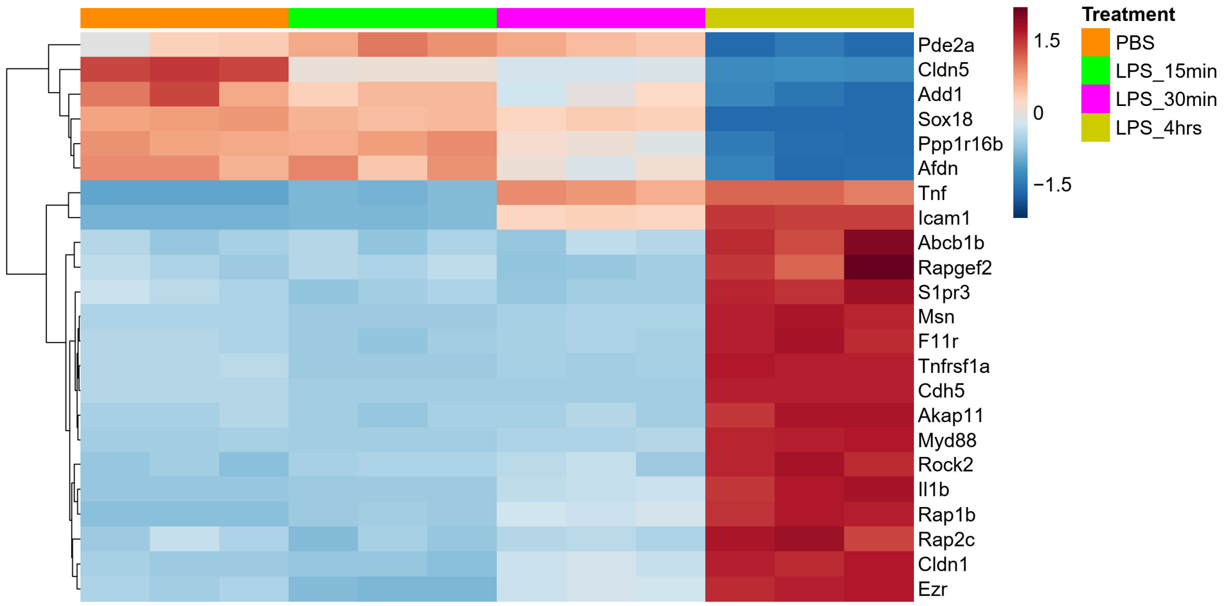

g

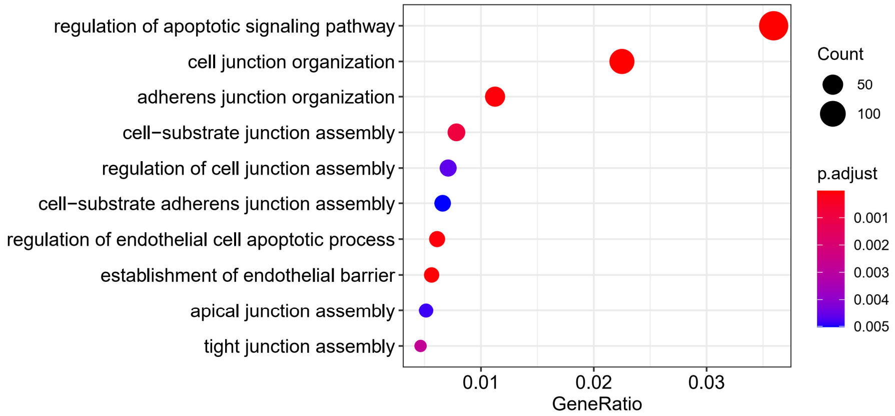

Supplementary figure. 2. Related to Figure. 2. Acute systemic inflammation rapidly affects the cerebral vessels during the initiation of SAE.

f. Heatmap showing the progression in significant genes belonging to gene ontology term establishment of endothelial barrier (GO:0061028), in the cerebral vessels from the LPS treatment time points 15 minutes, 30 minutes and 4 hours (significant genes (p.adj < 0.05 and LFC > 1.5) in LPS 4 hours compared to PBS. g. GO enrichment dot plots showing the number of genes affected and the top 10 enriched barrier related GO terms in the cerebral vessels at 4 hours LPS treatment compared to PBS.

Supplementary figure. 2 continued

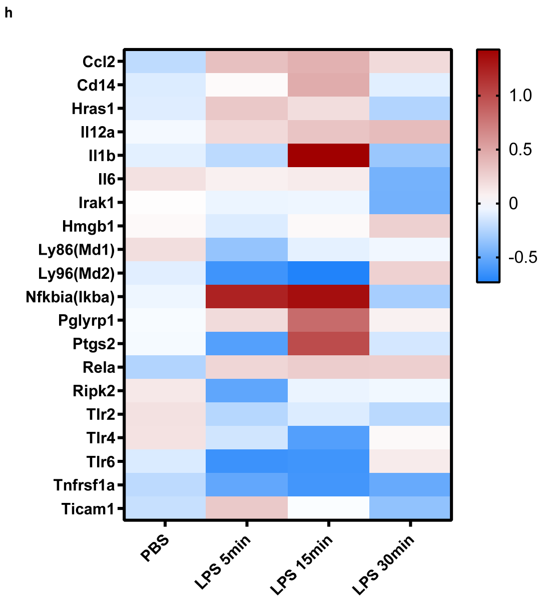

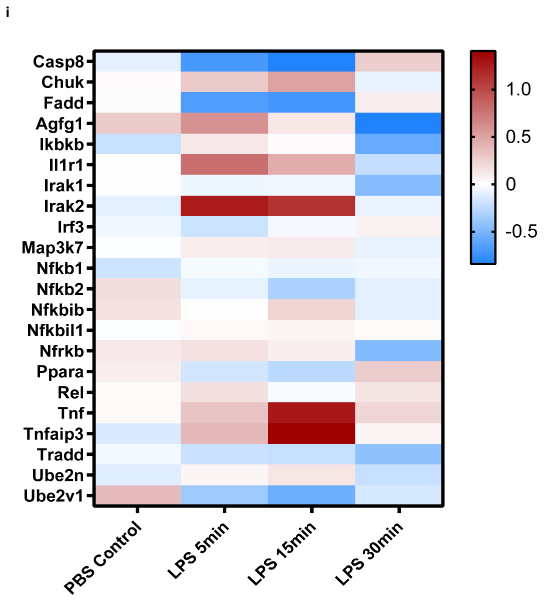

Supplementary figure. 2. Related to Figure. 2. Acute systemic inflammation rapidly affects the cerebral vessels during the initiation of SAE.

h-i. Heatmaps of the z-scores from the RT-qPCR on the cerebral vessels for the genes related to GO terms (h) response to lipopolysaccharide and (i) positive regulation of NF-κB signaling.

Supplementary figure. 3

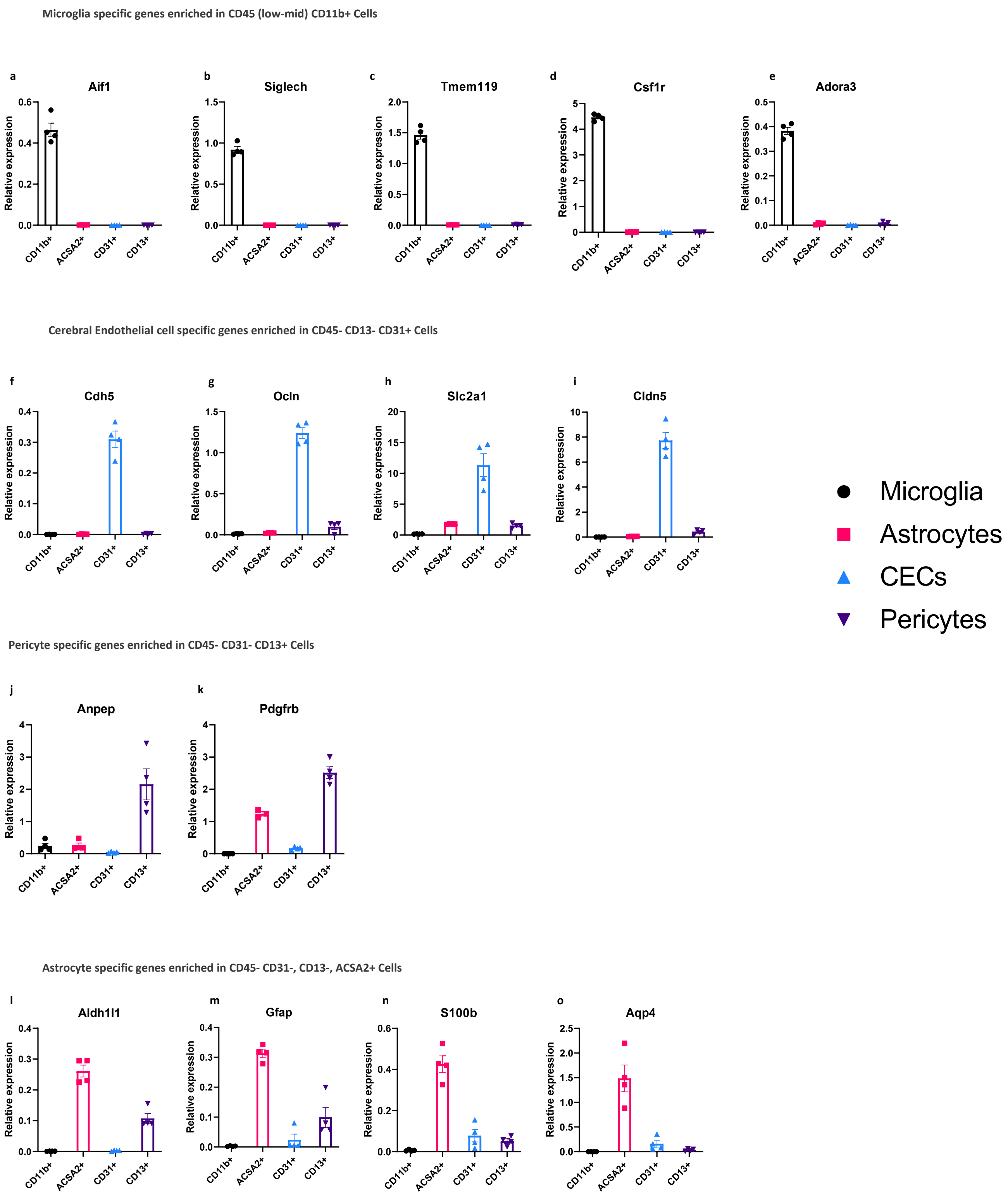

Supplementary figure. 3 Related to Figure 3. a-o. RT-qPCR analysis confirmed that the FACS sorting enriched for cells expressing cell type specific markers.

(a-e) CD45 low-mid CD11b+ sorting enriched for cells expressing microglial markers, (f-i) CD45- CD13- CD31+ sorting enriched for cells expressing cerebral endothelial cell markers, (j-k) CD45- CD31- CD13+ sorting enriched for cells expressing pericyte markers, and (l-o) CD45- CD31- CD13- O4- ACSA2+ sorting enriched for cells expressing astrocytic markers, (n=4 (males), Bars represent mean ± SEM)

Supplementary figure. 4

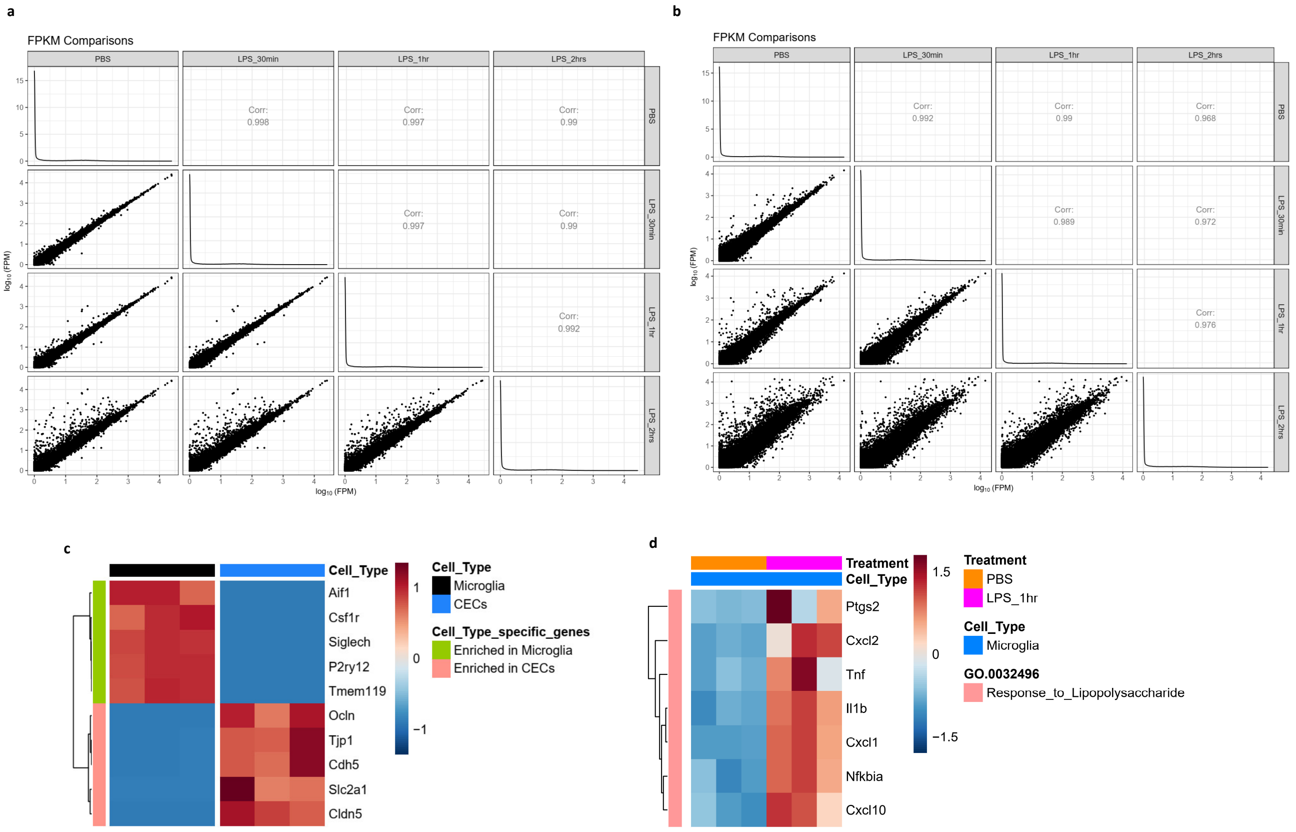

Supplementary figure. 4. Related to Figure 4. Temporal cell type specific transcriptional profiling of CECs and microglia following systemic inflammation

a-b Scatterplot matrices showing the FPKM distributions (histograms) and correlation (Corr) values of the RNA-seq data from (a) microglia and (b) CECs. c. Heatmap showing the enrichment of cell type specific marker genes in microglia and CECs. d. Heatmap showing the significant (p.adj < 0.05 and LFC > 1.5) genes belonging to gene ontology term response to lipopolysaccharide (GO:0032496) in microglia from the LPS treatment time point 1 hour.

Supplementary figure. 4 continued

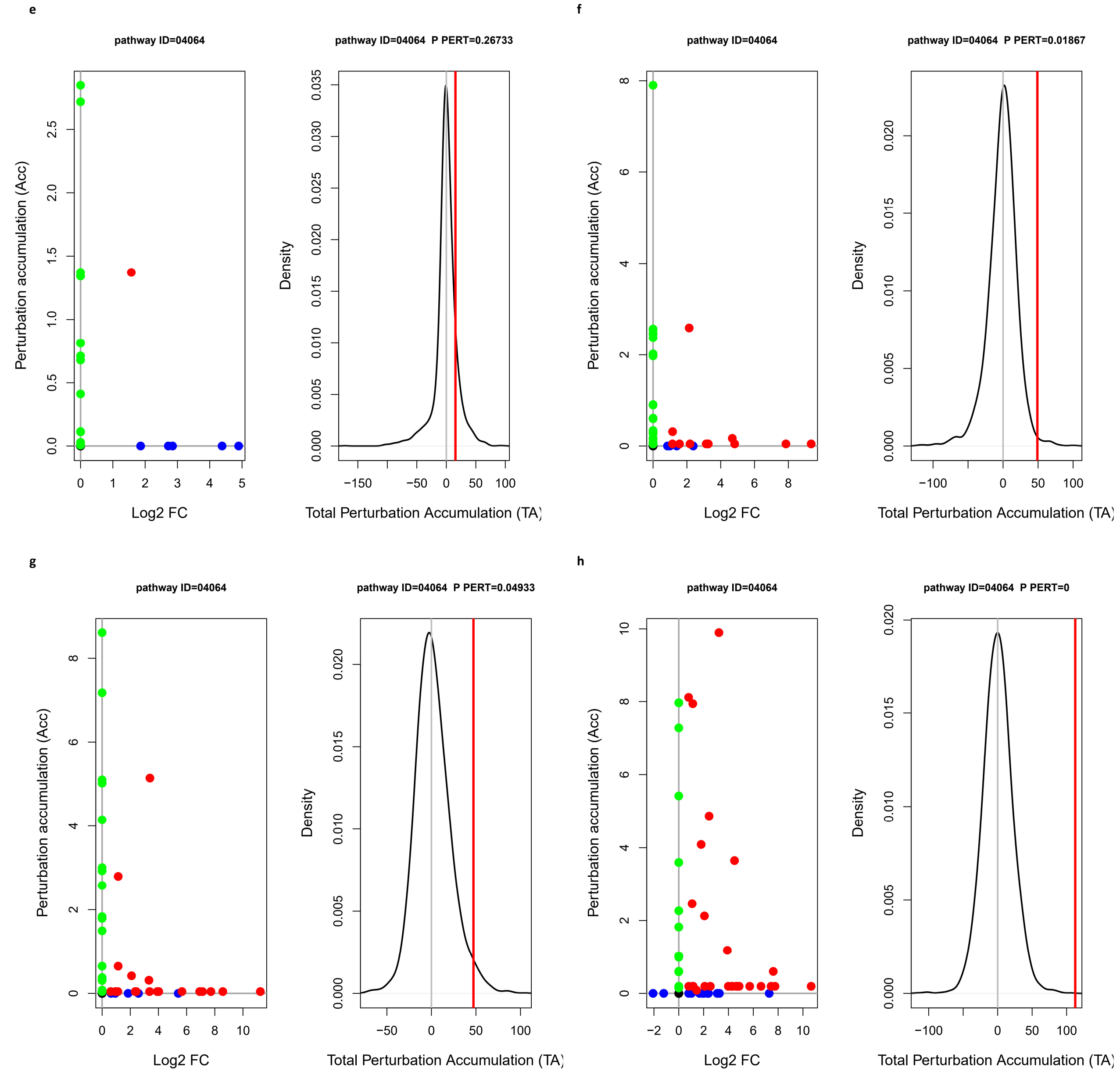

Supplementary figure. 4. Related to Figure 4. Temporal cell type specific transcriptional profiling of CECs and microglia following systemic inflammation

e-h Perturbation plots for NF-κB pathway (Kyoto Encyclopedia of Genes and Genomes (KEGG) ID mmu:04064) (e) in microglia from LPS treatment time point 1 hour compared to PBS (f) in CECs from LPS treatment time point 1 hour compared to PBS (g) in microglia from LPS treatment time point 2 hours compared to PBS. (h) in CECs from LPS treatment time point 2 hours compared to PBS. The perturbation of all genes in the pathway are depicted as a function of the log2 fold changes (left panel). Non differentially expressed genes are assigned 0 log2 fold-change. The null distribution of the net accumulated perturbation is also shown as a grey vertical line (right panel). The observed total accumulation (tA) with the actual data is shown as a red vertical line (right panel).

Supplementary figure. 4. continued

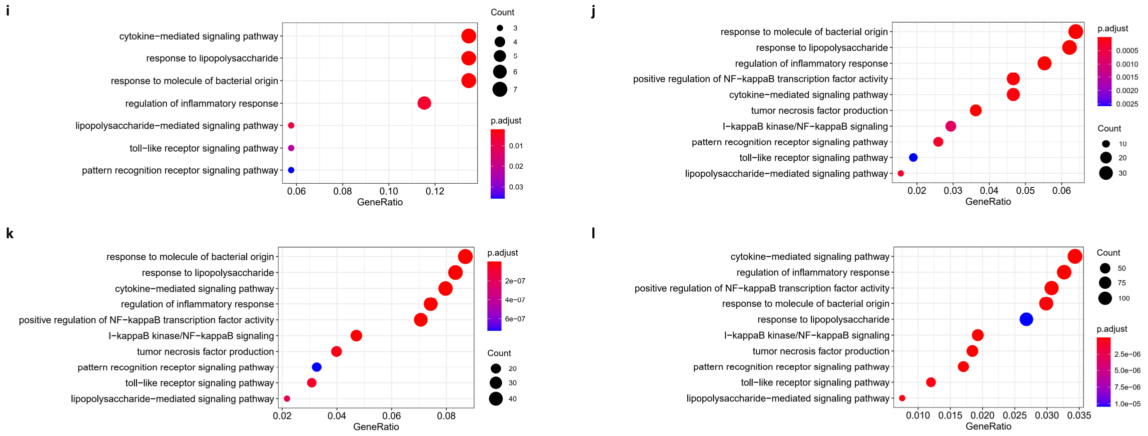

Supplementary figure. 4. Related to Figure 4. Temporal cell type specific transcriptional profiling of CECs and microglia following systemic inflammation

i-l. GO enrichment dot plots showing the number of genes affected in top 10 enriched inflammation related GO terms (i) in microglia from LPS treatment time point 1 hour compared to PBS (j) in CECs from LPS treatment time point 1 hour compared to PBS (k) in microglia from LPS treatment time point 2 hours compared to PBS (l) in CECs from LPS treatment time point 2 hours compared to PBS.

Supplementary figure. 4 continued

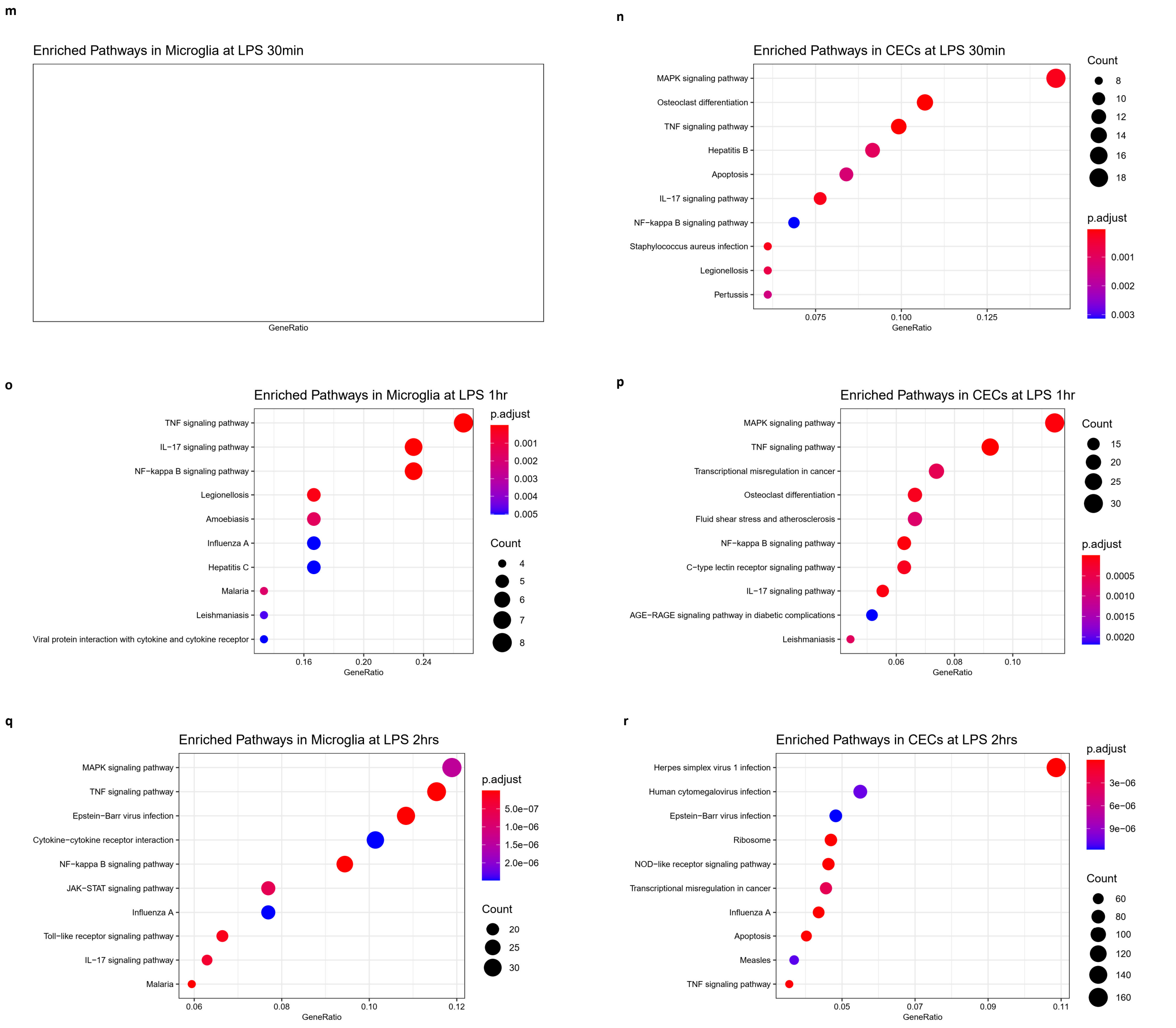

Supplementary figure. 4. Related to Figure 4. Temporal cell type specific transcriptional profiling of CECs and microglia following systemic inflammation

m-r. dot plots showing the enrichment of top 10 KEGG pathways and the associated number of genes affected (m) in microglia from LPS treatment time point 30 minutes compared to PBS (n) in CECs from LPS treatment time point 30 minutes compared to PBS (o) in microglia from LPS treatment time point 1 hour compared to PBS (p) in CECs from LPS treatment time point 1 hour compared to PBS (q) in microglia from LPS treatment time point 2 hours compared to PBS (r) in CECs LPS treatment time point 2 hours compared to PBS.

Supplementary figure. 4 continued

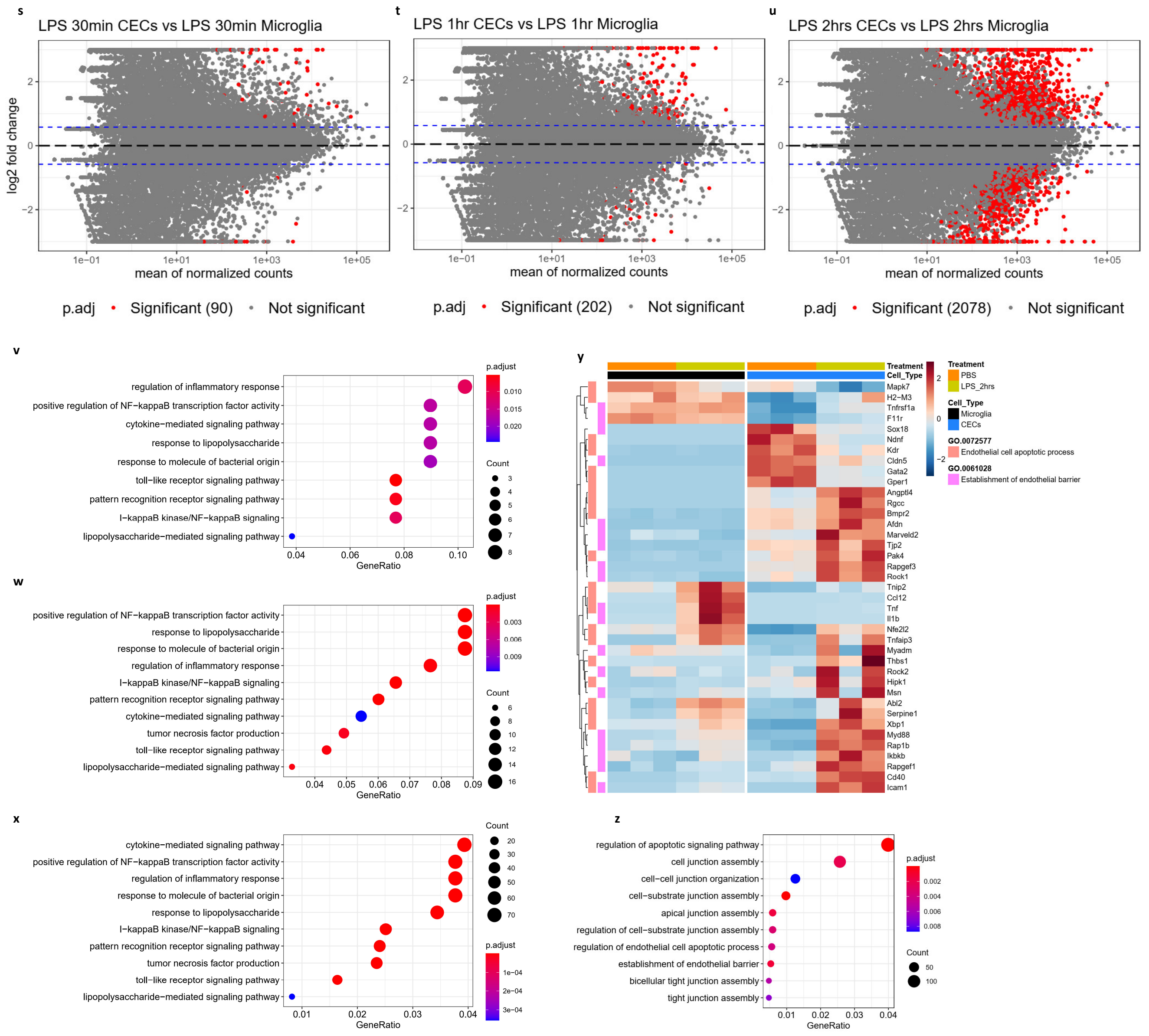

Supplementary figure. 4. Related to Figure 4. Temporal cell type specific transcriptional profiling of CECs and microglia following systemic inflammation

s-u. MA plots of the pairwise comparisons after likelihood ratio test (LRT) using reduced model in DESeq2, in CECs compared to microglia at timepoints (s) 30 minutes, (t) 1 hour and (u) 2 hours. Log fold changes (LFCs) are plotted against the mean of normalized counts to determine the variance between two treatments in terms of gene expression. Red nodes on the graph represent statistically significant data points i.e. p.adj < 0.05 and LFC > 1.5.Gray nodes are data points that are not statistically significant. Numerical values in parentheses for the significant legend indicate the number of genes that meet the prior condition. Dashed lines indicate the cutoff LFC values. v-x. GO enrichment dot plots showing the number of genes affected in top 10 enriched inflammation related GO terms (v) at LPS 30 minutes in CECs compared to LPS 30 minutes microglia (w) at LPS 1 hour in CECs compared to LPS 1 hour microglia (x) at LPS 2 hours in CECs compared to LPS 2 hours microglia, y. Heatmap showing the changes in significant genes belonging to gene ontology terms establishment of endothelial barrier (GO:0061028) and endothelial cell apoptotic processes (GO:0072577), in microglia and CECs at LPS treatment time point 2 hours (significant genes (p.adj < 0.05 and LFC > 1.5) in CECs LPS 2 hours compared to CECs PBS). z. GO enrichment dot plot showing the number of genes affected and the top 10 enriched endothelial barrier related GO terms in the CECs at LPS 2 hours compared to PBS.

Supplementary figure. 5

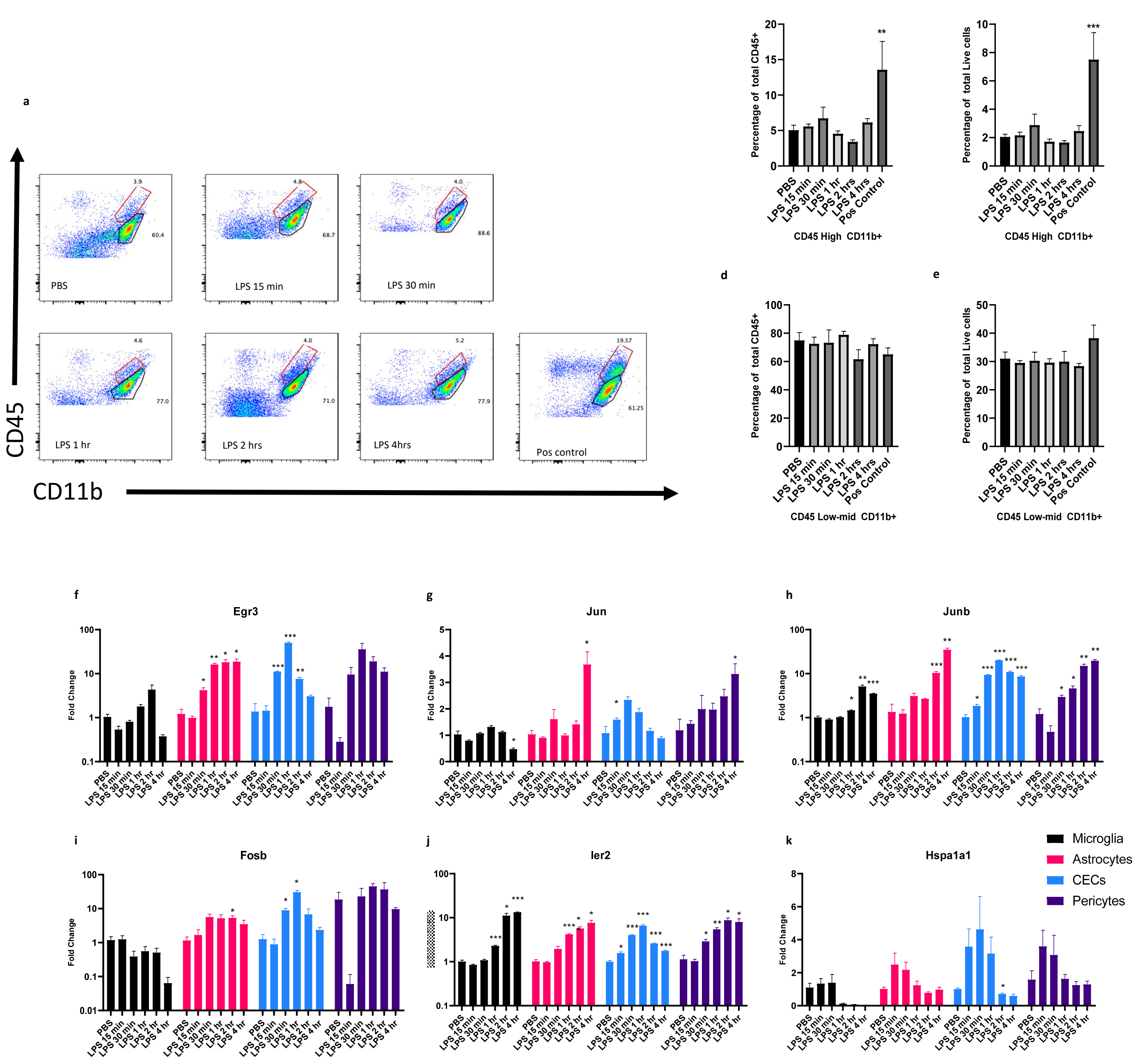

Supplementary figure. 5. related to Figure.5. Cerebral endothelial cells are the earliest to respond to the peripheral inflammation in the CNS.

a. Flow cytometry gating strategy of brain CD45 high CD11b+ (indicated by red gates) and CD45 low-mid CD11b+ (indicated in brown gates) myeloid cells from the mice injected with 10mg/kg LPS and sacrificed at the indicated time points, mice injected with LPS (1mg/kg) once daily for four days consecutive days and sacrificed 24 hours after the last injection are used as positive control (Pos control) . b-e. Quantitation of brain (b-c) CD45 high CD11b+ and (d-e) CD45 low-mid CD11b+ as a percent of total CD45 and total live cells. f-k. Time course of mRNA fold change in microglia, astrocytes, CECs and pericytes isolated from the brains of mice injected with 10mg/kg LPS f. *Egr3*, g. *Jun*, h. *Junb*, i. *Fosb*, j. *Ier2*, k. *Hspa1a1*. Graphs depict mean ± SEM, n= 4 (males)each group, Two-way ANOVA with Dunnett post-hoc test, *p<0.05, **p<0.01, ***p<0.001.
